# Entropy-Driven Cellulosic Elastomer Self-Assembly for Mechanical Energy Harvesting and Self-Powered Sensing

**DOI:** 10.1007/s40820-025-02054-y

**Published:** 2026-01-21

**Authors:** Pinle Zhang, Yingping He, Huancheng Huang, Neng Xiong, Xinyue Nong, Xinke Yu, Shuangfei Wang, Xinliang Liu

**Affiliations:** Guangxi Key Laboratory of Clean Pulp & Papermaking and Pollution Control, School of Light Industry and Food Engineering, Guangxi University, Nanning, 530004 People’s Republic of China

**Keywords:** Cellulosic elastomers, Entropy-driven self-assembly, Mechanoelectric conversion, Self-powered sensing

## Abstract

The rapid advancement of flexible electronics technology has placed higher demands on the structural design and performance regulation of elastic materials. Cellulosic elastomers, with their biodegradability, renewability, and tunability, emerge as ideal candidate materials. Entropy-driven self-assembly promotes the spontaneous formation of ordered structures, serving as a crucial pathway for optimizing cellulose elastomer properties. However, the structure–property relationship between the self-assembled ordered structures of cellulose elastomers and their mechanical and electrical properties remains insufficiently explored. It hinders the expansion of their applications in electronic devices. This paper systematically reviews the structure–property regulation mechanisms of self-assembled cellulosic elastomers from an entropy-driven perspective. It elucidates the application principles and performance optimization strategies for mechanical energy harvesting and self-powered sensing, while also exploring the challenges and prospects for performance enhancement. This work provides a reference for the development of self-assembled cellulosic elastomers in the field of energy devices.

## Highlights

- It systematically discusses the contribution of entropy-driven approaches to the design of self-assembled structures and performance regulation in cellulosic elastomers.
- This review systematically examines design strategies for ordered self-assembled structures in cellulosic elastomers and investigates their structure-property relationships.
- It presents a comprehensive review of performance design strategies for self-assembled cellulosic elastomers across mechanical and electrical domains, focusing on electromechanical conversion and self-powered sensing applications.

## Graphical abstract

**Figure:**
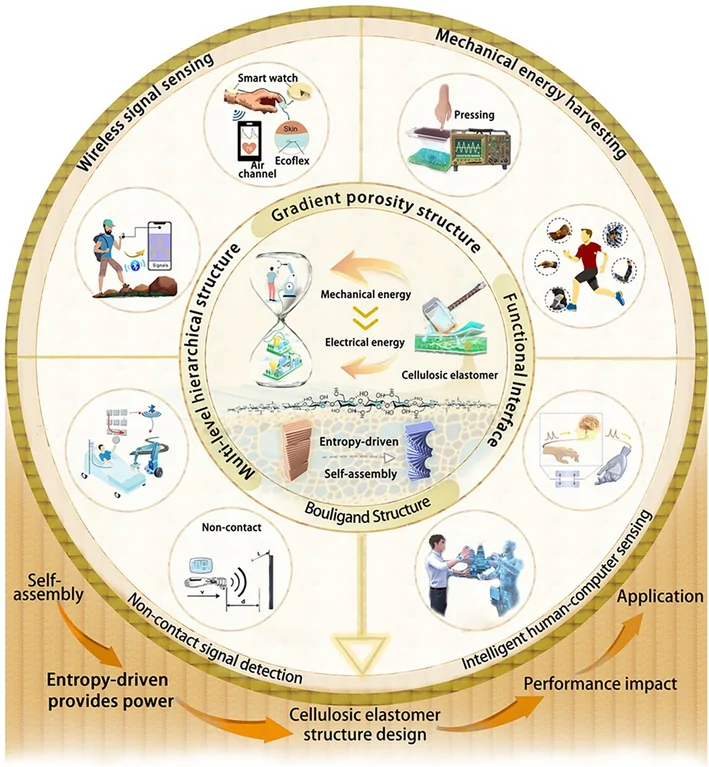

## Introduction

The development of flexible electronic devices represents a major challenge in the field of materials science and technology. Originating from fundamental theoretical exploration, this technology not only promises to drive diverse innovative applications in advanced electronic products such as micro-energy harvesters [1, 2], electronic skin [3, 4], flexible displays [5], biosensors, and wearable electronics [6, 7], but also imposes higher demands on the structural design and performance regulation of its core material—elastomers [8–11]. Stretchable flexible electronics can cover arbitrary curved surfaces and moving components (e.g., robotic arm joints, medical bandages) [12–14], greatly expanding their application scenarios and making flexible elastomers the key materials for achieving this goal. In emerging energy fields, triboelectric nanogenerators (TENG) [15–20], piezoelectric nanogenerators (PENG) [21–23], and dielectric elastomer generators (DEG) [24] have become vital sources of clean energy, demonstrating immense potential particularly in low-frequency micro-energy harvesting and self-powered sensor-energy-harvesting integration [25]. Elastomer materials demonstrate unique advantages in novel wearable energy and sensing devices due to their excellent mechanical properties, biocompatibility, high sensitivity, and signal-to-noise ratio [26]. Recent research has continuously focused on enhancing the electromechanical conversion efficiency of elastomer energy devices through various enhancement strategies and expanding their applications in self-powered sensing.

However, current elastomer energy harvesting and sensing technologies still face multiple challenges: on one hand, traditional elastomer materials struggle to balance high electromechanical performance with environmental sustainability, as their non-degradable nature contributes to the growing electronic waste problem [27–29]; on the other hand, existing material systems often fail to simultaneously meet the comprehensive requirements of stretchability, thermal stability, and biocompatibility while maintaining high sensitivity and signal-to-noise ratio [29–34]. These limitations severely constrain the further application of flexible electronic devices in terms of sustainability and long-term reliability. Against this backdrop, cellulose-based elastomers demonstrate unique advantages distinct from traditional synthetic materials: Its performance advantages stem from its multiscale structural features, including molecular chains, supramolecular chains, and macroscopic fibers. Hydroxyl groups on cellulose molecular chains enhance mechanical properties through hydrogen bonding and electrostatic interactions [35]. They readily accept grafting modifications to introduce dynamic covalent bonds, thereby improving mechanical strength and self-healing capabilities [36]. Within cellulose’s supramolecular structure, crystalline regions provide rigidity and strong polarity, while amorphous regions confer flexibility and impact resistance [37–40]. At the macroscale, the order of cellulose molecules and aggregates significantly influences electrical properties [41], stiffness [42], elasticity [43], and surface energy [44]. Its networks effectively reduce internal defect density, enabling synergistic optimization of reduced dielectric loss, enhanced breakdown strength, and improved mechanical properties [45, 46].

Ordered structures form spontaneously through self-assembly processes, which minimize the system’s free energy by increasing entropy—the key driving force of self-assembly [47]. Thus, entropy is central to the formation of self-assembled structures. Leveraging the bonding properties of cellulose and environmental conditions (temperature, pH, pressure, etc.) [48, 49], self-assembled structures at different hierarchical levels can be directionally constructed, enabling precise control over material properties. At the microscopic level, entropy-driven nanoscale chiral ordered structures not only promote efficient stress dispersion but also enhance dipole orientation, synergistically improving both mechanical properties and dielectric response [50]. At the macroscopic level, gradient structures and multi-level biomimetic architectures formed under entropy regulation buffer external mechanical forces and suppress charge dissipation, thereby enhancing mechanical stability and electrical energy conversion efficiency. Furthermore, such structures confer exceptional conformability and wear comfort to the material, significantly enhancing its sensing stability and environmental adaptability in wearable applications. Consequently, the entropy-driven self-assembly mechanism provides a theoretical foundation for overcoming the performance limitations of cellulose elastomers, enabling the construction of structures with biomimetic properties and energy conversion capabilities [51–53]. This approach not only underpins the structural design of mechanical-to-electrical energy conversion materials but also establishes a material foundation for developing high-precision, multifunctional power generation devices and sensors.

**Fig. 1:**
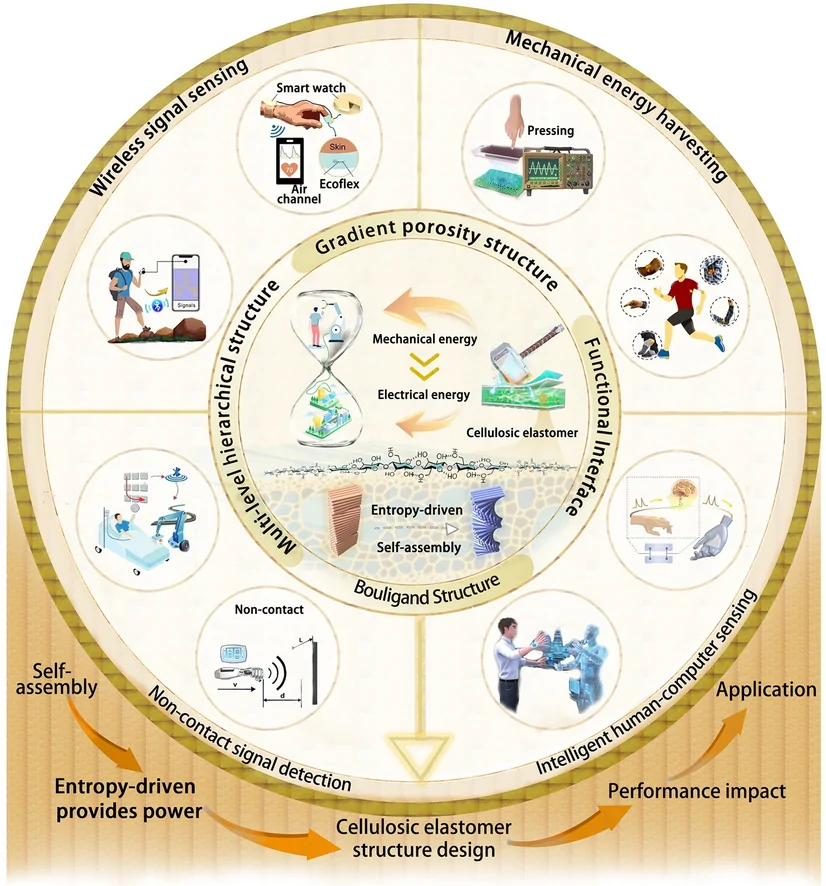
Entropy-driven self-assembly of cellulose-based elastomeric materials and their applications in energy harvesting and self-powered sensing

Currently, numerous studies on self-assembled cellulosic elastomers have been reported. For instance, some research investigates the self-assembly of cellulose into chiral phase arrays, designed for information encryption, or into skin-like gradient structures for wearable electronic skin devices. Particularly in the field of optics, the development of chiral phase array structures has been well documented [54–57]. However, existing research generally lacks sufficient attention to the critical role of entropy-driven mechanisms in the self-assembly process. Systematic summaries of the molecular dynamic characteristics of self-assembly in mechanical and electrical fields remain inadequate. The regulatory mechanisms governing the “structure–property” relationship in entropy-driven cellulosic elastomer self-assembly have not been thoroughly explored. Consequently, material design is still heavily dependent on empirical exploration, thus precluding precise control over material performance. This limitation constrains the effective application of cellulosic elastomer materials in mechanical energy harvesting and self-powered sensing. This paper provides a systematic review of research progress in entropy-driven cellulosic elastomer self-assembly across mechanical and electrical domains. It first introduces the molecular structural characteristics of cellulosic elastomers and their entropy-driven self-assembly properties, systematically elucidating bonding types, structural regulation mechanisms, and performance optimization pathways. Subsequently, it delves into the principles of electromechanical conversion and its applications in piezoelectric power generation, triboelectric nanogenerators, and dielectric elastomer power generation. Finally, it summarizes compatibility challenges between elasticity and electromechanical conversion performance, bottlenecks in mass production, and outlines future trends toward multimodal self-powered integrated systems (Fig. 1). This work aims to provide theoretical foundations and design insights for innovative, efficient energy-harvesting technologies and self-powered sensors, driving breakthrough applications of next-generation flexible electronics in complex scenarios.

## Entropy-Driven Self-Assembly of Cellulosic Elastomers

**Fig. 2:**
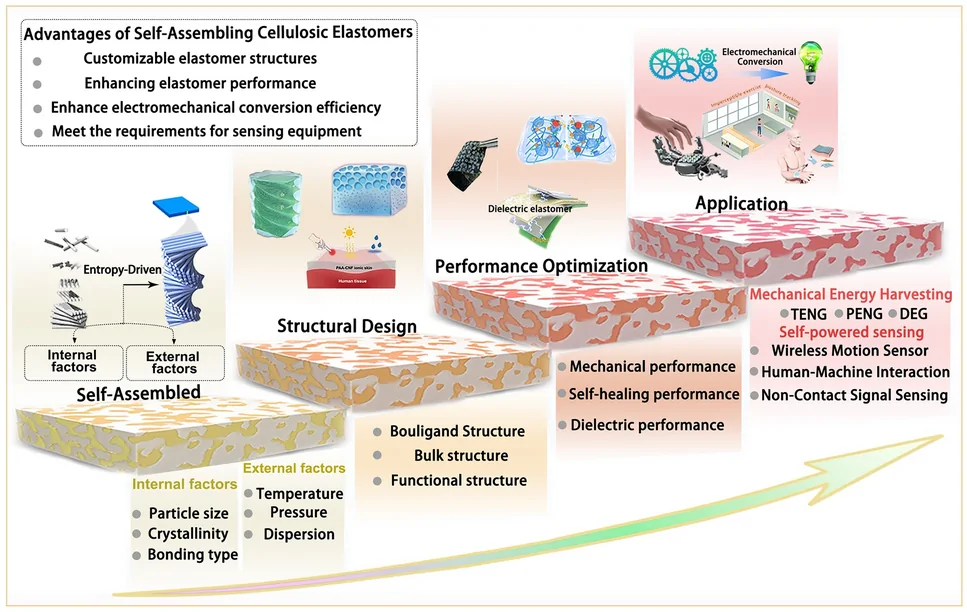
Entropy-driven design and applications of self-assembled cellulosic elastomers for electromechanical energy conversion and self-powered sensing

The spontaneous evolution of a system is primarily driven by an increase in entropy sufficient to overcome unfavorable or only marginally favorable enthalpy changes, leading to a net decrease in Gibbs free energy. This thermodynamic principle enables the system to evolve toward a macroscopic state encompassing more accessible microstates. In the context of this study, we clearly delineate the role of entropy-driven processes in the formation of ordered nanostructures from molecular building blocks. Specifically, we emphasize how the increase in conformational entropy from flexible linkers, or the gain in translational entropy from released ions, serves as the primary driving force behind the observed structural ordering. This mechanism distinguishes our system from those governed predominantly by enthalpy-dominated interactions. Building upon this entropy-driven framework, we demonstrate the design of self-assembled cellulosic elastomers under functional entropy-favorable conditions. By leveraging hydrogen bonds, weak interactions, and non-dynamic covalent bonds within the system, we achieve customizable control over the structural properties of the elastomers. This strategy effectively addresses the performance requirements of elastomer materials in energy harvesting and self-powered sensing applications. The proposed approach holds significant potential for the development and rational design of new energy materials, offering clear guidance for enhancing electromechanical conversion efficiency and electronic sensing performance, as illustrated in Fig. 2.

### Molecular Structural Characteristics of Cellulosic Elastomers

**Fig. 3:**
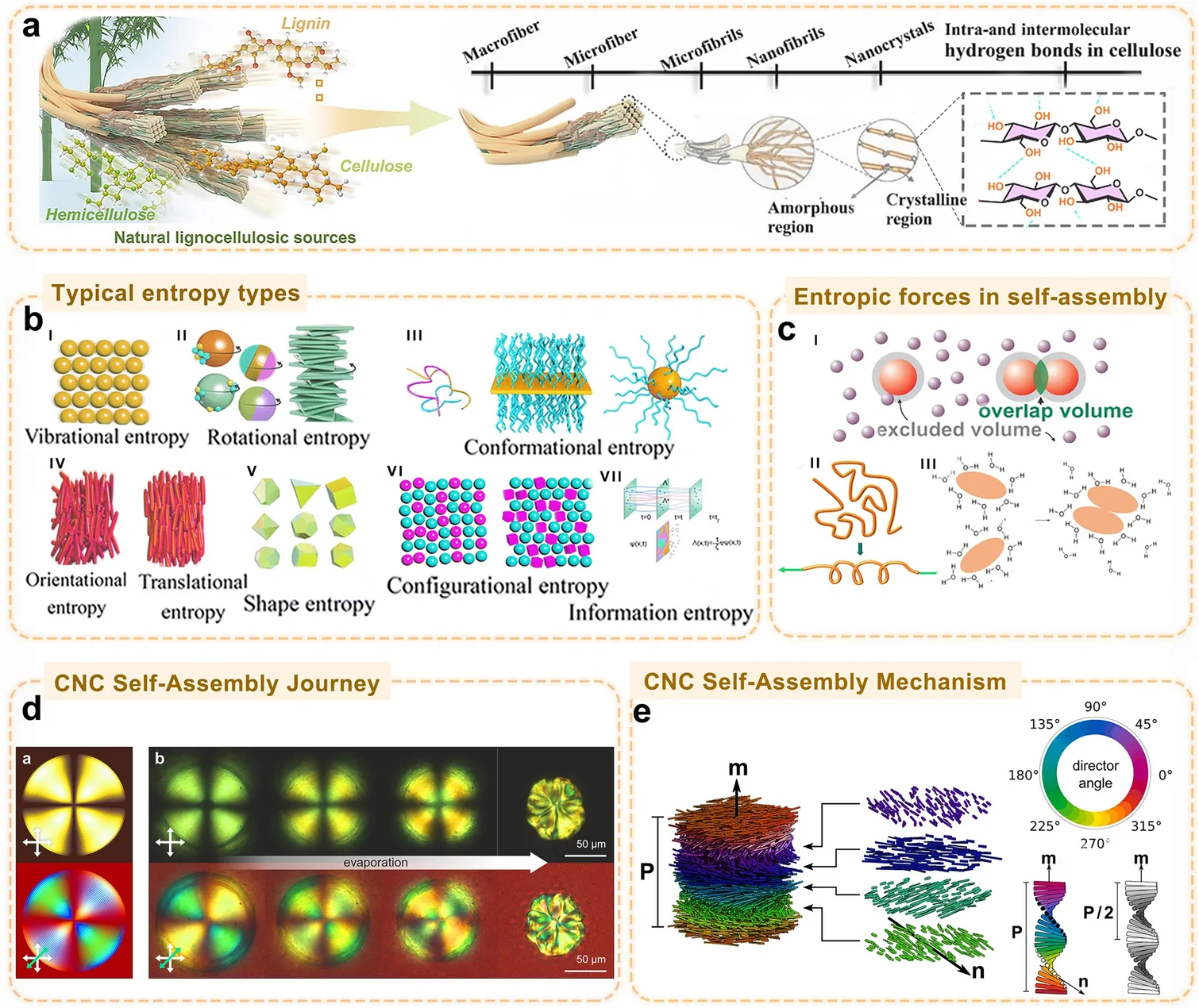
Structural features of cellulose molecules. **a** Cellulose hierarchical structure. Reproduced with permission from Ref. [81], Copyright 2025 American Chemical Society. **b** Types of entropy (I vibrational entropy, II rotational entropy, III orientational and translational entropy, IV shape entropy, V conformational entropy, VI configurational entropy, and VII information entropy). Reproduced with permission from Ref. [70], Copyright 2023 Royal Society of Chemistry. **c** Entropy-driven self-assembly mechanism (I dissipative force, II elastic force, and III nonpolar surface). Reproduced with permission from Ref. [71], Copyright 2006 Rockefeller University Press. **d** Entropy-Driven Self-Assembly Processes. Reproduced with permission from Ref. [82], Copyright 2019 Nature Publishing Group. **e** Entropy-Driven Self-Assembly Mechanism. Reproduced with permission from Ref. [83], Copyright 2021 American Chemical Society

Cellulose, a renewable, biodegradable, and environmentally friendly material [58–60], is a linear polymer composed of glucose units linked by β-1,4-glycosidic bonds [61]. This structure contrasts with the highly rigid chains of polyvinylidene fluoride (PVDF) and the highly flexible molecular chains of polydimethylsiloxane (PDMS) [62, 63]. Cellulose comprises crystalline regions (where molecular chains are tightly packed) and amorphous regions (where chain structures are loosely arranged), exhibiting both high stiffness and extensibility. Benefiting from this characteristic, the cellulose crystal-amorphous equilibrium, chain stiffness, and hydrogen bond network collectively regulate configurational entropy, thereby determining the material’s structural order and diversity (Fig. 3a) [15, 64–67]. Specifically, the loosely arranged amorphous regions exhibit weaker interactions like hydrogen bonds [68], resulting in higher system entropy. Upon external stimulation, amorphous cellulose chains reorganize through self-assembly to reduce the system’s free energy. This process is driven by entropy increase, meaning the system achieves macroscopic order by expanding the number of microscopic states. Crystalline regions exhibit lower conformational entropy due to ordered molecular packing, while amorphous regions possess higher entropy owing to greater molecular freedom. The ratio between these regions can be dynamically regulated by external conditions like temperature and pressure, thereby influencing the material’s macroscopic properties. Chain stiffness directly constrains conformational freedom [69]: highly rigid chains exhibit low entropic values, while flexible chains display higher entropy. Balancing mechanical behavior and entropy change is achieved by modulating chain flexibility. The strength of hydrogen bond networks determines the degree of intermolecular order. Strong hydrogen bonds form ordered aggregates that reduce entropy, while weak hydrogen bonds maintain higher conformational entropy. These three mechanisms synergistically regulate conformational entropy, providing key theoretical guidance for achieving ordered structural design and functional optimization in materials.

### Entropy-Driven Contribution to the Self-Assembly of a Cellulosic Elastomer

Entropy-driven processes play a pivotal role in material structure design through self-assembly mechanisms in nature. These processes accelerate the increase in a system’s total entropy—comprising vibrational, rotational, and configurational entropy (Fig. 3b)—by promoting spontaneous molecular rearrangements under thermodynamic equilibrium [70]. In cellulose systems, the dominant entropy contributions depend on specific physicochemical processes and system conditions. During dispersion, translational entropy and interfacial entropy typically prevail; in mechanical property regulation and hydration, conformational entropy and interfacial entropy play more significant roles. Different types of entropy are interrelated, collectively determining the structure and properties of cellulose systems. The self-assembly of cellulose elastomers exemplifies such entropy-driven dynamic equilibrium. In these systems, entropic forces—such as dissipative forces [71], elastic forces, and nonpolar surface interactions (Fig. 3c)—guide the formation of ordered structures by maximizing total entropy in a controlled manner. From a thermodynamic perspective, the system’s free energy G is jointly determined by enthalpy H and entropy S, highlighting how entropy generation governs assembly pathways [72].

1 G=H-TS

Temperature T represents the tendency for free energy G to minimize toward thermodynamic equilibrium. Typically, self-assembling systems reduce free energy by increasing entropy. This reduction can occur through two pathways: increasing entropy (potentially increasing the number of possible microstates within the system) or decreasing enthalpy (lowering the internal energy of the system). During self-assembly, these entropy-increasing pathways can drive the formation of ordered structures within the system. Hydrophobic interactions exemplify entropy-driven mechanisms, their strength intensifying with rising temperatures. Quantitatively, hydrophobic units form repulsive volume regions in water, causing water molecules to rearrange around them and thereby incurring an entropy cost. This entropy change can be quantified by the reduction in spatial conformational volume afforded by hydrogen bond formation: small hydrophobic units diminish the available conformational space for water molecules, lowering the system’s number of microstates. However, when hydrophobic units self-assemble, the system achieves a net entropy increase by reducing repulsive volume and releasing ordered water molecules, thereby driving assembly [73, 74]. The spontaneous self-assembly of cellulose molecular chains in solution follows the principle of entropy maximization [75, 76], where the system achieves thermodynamically stable conformations by minimizing Gibbs free energy. When cellulose nanofibers (CNF) or cellulose nanocrystals (CNC) are dispersed in solvent, structural color changes are observable under cross-polarized light, arising from the formation of ordered solvation layers between the surface-abundant hydroxyl groups and solvent molecules [77]. As water evaporates, the system’s entropy decreases. To compensate for this entropy loss, cellulose molecules spontaneously assemble into higher-order structures (e.g., nematic liquid crystals), restoring entropy equilibrium at a larger scale and yielding brighter colors [78] (Fig. 3d). To more intuitively illustrating entropy-driven self-assembly of ordered cellulose structures, the CNC suspension is depicted as a helical configuration. The guiding field n(r) rotates periodically along an axis termed the helical axis. The resulting alignment can be described as a stack of continuously rotating nematic alignment layers with infinitesimal thickness (Fig. 3e). The helical axis can be characterized by an additional nonpolar unit vector m, ensuring the orienter remains perpendicular to the helical axis (n ⊥ m). This helical structure reveals that precise programming of macroscopic properties in chiral soft matter can be achieved by balancing chiral interactions with elastic forces.

In supramolecular polymer systems, supramolecular polymers exist in a state of thermodynamic equilibrium, with their assembly adjustable through external stimuli such as temperature. Entropy changes manifest here as a synergy between steric volume effects and secondary interactions: in the liquid crystalline state, entropy effects (such as steric volume) combine with weak interactions to facilitate the formation of one-dimensional polymeric structures [79]. Therefore, entropy-driven force is the core driving force for the self-assembly of cellulosic elastomers. It promotes the spontaneous ordered arrangement of molecules by maximizing the system’s entropy value. Cellulose nanoparticles form mesoscopic ordered structures in solvents by reducing the excluded volume and balancing the entropy-enthalpy relationship [80]. This entropy-driven self-assembly mechanism confers dynamically responsive properties to materials, enabling precise modulation through external stimuli such as temperature and ionic strength. The process facilitates energy-efficient and fully reversible structural reconfiguration, thereby establishing a fundamental framework for the rational design of intelligent materials.

### Factors Affecting Entropy-Driven Self-Assembly of Cellulosic Elastomer

The factors influencing the entropy-driven process of cellulose elastomers can be broadly categorized into two types (Table 1)—internal factors (molecular scale, crystallinity, functional groups) and external factors (temperature, pressure, solvent environment). Cellulose scale, cellulose crystallinity, and cellulose functional groups are key components within its own structure [84]. At the cellulose scale, self-assembly frequently occurs at the micrometer and nanometer scales. The minute dimensions of cellulose fibers create more favorable conditions for colloidal Brownian motion within self-assembly systems, endowing them with high translational and rotational entropy values that promote the initiation of self-assembly [85, 86]. Furthermore, high aspect ratio nanofibrils exhibit a substantial volume repulsion effect that significantly enhances the driving force of orientation entropy. This allows the system to achieve substantial translational entropy gains at lower concentrations by sacrificing a small amount of orientation entropy, thereby markedly lowering the isotropic-nematic transition temperature. Conversely, this effect leads to an increase in the transition temperature.

**Table 1:** Factors influencing entropy-driven self-assembly of cellulose elastomers

Factor Category	Influencing Factors	Effect on Entropy Change (ΔS)	Effects on Self-Assembly Driving Forces	References
Internal factors	Cellulose particle size	The smaller the initial particle size, the greater the number of ordered water molecules bound, resulting in a larger increase in entropy (ΔS) after assembly	Smaller particle sizes confer greater mobility to the system, promoting the occurrence of self-assembly	[85, 86]
	Crystallinity	Low crystallinity can confine a large amount of ordered water, leading to a significant increase in entropy during assembly, whereas high crystallinity exhibits only a limited increase in entropy	High-crystallinity particles tend to assemble spontaneously. The assembly behavior of low-crystallinity particles is dominated by other interactions	[87]
	Functional group	Aggregation occurs through electrostatic attraction, hydrogen bonding, covalent bonding, etc., reducing entropy, while electrostatic repulsion increases entropy (ΔS)	Electrostatic repulsion creates conditions for ordered assembly, while hydrogen bonds and covalent bonds jointly determine the diversity of interactions	[90]
External factors	Temperature	Increasing temperature amplifies the contribution of the entropy increase term (TΔS)	Moderate heating significantly enhances assembly driving forces and kinetics. Excessive heating disrupts the formed ordered structures	[91]
	Pressure	High pressure can disrupt the hydrogen bond network between cellulose and water, thereby increasing entropy gain (ΔS)	Provide compressive driving force for assembly and induce reorganization along specific pathways	
	Solvent environment	Low pH/high ionic strength weakens electrostatic repulsion, promoting aggregation and reducing entropy (ΔS)	Low pH/high ionic strength enhances assembly driving force, while high pH/low ionic strength enhances stability	

Cellulose crystallinity determines its morphology: highly crystalline cellulose forms rod-like particles that readily spontaneously assemble into entropy-driven chiral structures to maintain rotational entropy and release solvent degrees of freedom. In contrast, low-crystallinity particles hinder chiral structure formation [87, 88]. This arises because the crystallinity index governs the efficiency of this entropy-driven process by regulating the synergy of particle interactions: a high crystallinity index ensures uniformity in size, shape, and charge distribution, enabling synergistic ordered arrangement at low concentrations and thus significantly lowering the isotropic-nematic transition concentration; Conversely, the multiscale disorder introduced by low-crystallinity indices severely impedes entropy-driven ordering, leading to a marked increase in transition concentration or even suppression of phase transition.

Cellulose functional groups significantly influence entropy-driven self-assembly, primarily through their bonding types within the system. The abundant hydroxyl groups in cellulose molecular structures impart negative charge to cellulose particles. Hydrogen bonds and electrostatic interactions precisely guide assembly processes by finely regulating the dynamic equilibrium between “solvent entropy gain and solute entropy loss.” Specifically [89]: Electrostatic repulsion acts as a competitive regulatory factor. By establishing an energy barrier that mutually constrains the entropy-driven force (such as the dissipative force), it prevents disordered aggregation of solute particles while ensuring that the loss of configurational entropy during the ordering process is effectively compensated by the significant gain in solvent entropy. This guides the formation of thermodynamically stable ordered structures [90]. Concurrently, hydrogen bonding acts as a cooperative regulatory factor. Leveraging its unique directionality and specificity, it provides critical structural guidance and binding sites for the assembly driven by entropy. This synergistic interaction with the entropy-driven force jointly determines the final configuration and symmetry of the superstructure. The synergistic and competitive mechanisms between these two forces collectively maintain the delicate equilibrium between solvent entropy and solute entropy, forming a crucial foundation for achieving controllable self-assembly. Furthermore, the relatively active C_6_ hydroxyl groups on cellulose facilitate grafting of other functional groups (carboxyl, sulfate, sulfonate, etc.), enabling additional interactions between cellulose molecules (e.g., hydrophobic interactions, conjugation effects). This further enhances the diversity of self-assembled cellulosic elastomer structures.

Another category involves external factors such as temperature, pressure, and solvation environment of the cellulose medium. While elevated temperatures increase molecular thermal motion entropy, they may disrupt ordered structures. Moderate heating, however, can provide energy to overcome energy barriers, promoting the formation of entropy-driven liquid crystal phases. Increased pressure generally restricts system degrees of freedom and reduces entropy, promoting the formation of compact ordered structures. However, under specific conditions, compression can induce rearrangements that yield entropy gains. The ionic strength, hydrophobic effects, and polarity of dispersants within the solvation environment play crucial roles. Enhanced ionic strength can shield electrostatic repulsion, facilitating entropy-driven ordered assembly, but excessive shielding leads to aggregation. The hydrophobic effect of water can significantly release molecular entropy to drive assembly, while changes in dispersant polarity may alter the equilibrium between hydrophobic interactions and hydrogen bonding [91].

In cellulose entropy-driven self-assembly, multiple parameters synergistically regulate its multi-level structural evolution [92, 93]: particle size, crystallinity, hydrogen bonding/electrostatic interactions, temperature, pressure, and solvent environment. Small particle size provides high translational entropy to drive free molecular chain motion [94], while high crystallinity confers a rigid framework supporting ordered arrangement. The equilibrium between hydrogen bonding and electrostatic interactions both guides specific assembly and maintains structural stability. Temperature and pressure, as external enabling factors, activate molecular motion and induce densification and ordering, respectively. The solvent environment ultimately fixes the network topology by modulating interaction strengths. This multiscale synergy constructs a three-dimensional network architecture featuring ordered, densely interconnected structures. By optimizing stress transmission pathways and charge transport channels, it holds potential for enhancing the material’s piezoelectric response, triboelectric output, and strain sensing performance.

Thus, entropy-driven self-assembly in cellulosic elastomers exhibits remarkable structural tunability, arising from the synergistic interplay between intrinsic properties (scale, crystallinity, functional groups) and environmental conditions (temperature, pressure, solvation). These factors enable controlled preparation of multiscale structures from nanoscale to macroscale by regulating the system’s entropy-enthalpy balance and diverse interactions, providing an effective and controllable pathway for designing ordered material structures.

### Cellulosic Composite Elastomer Self-Assembled by Entropy-Driven

**Fig. 4:**
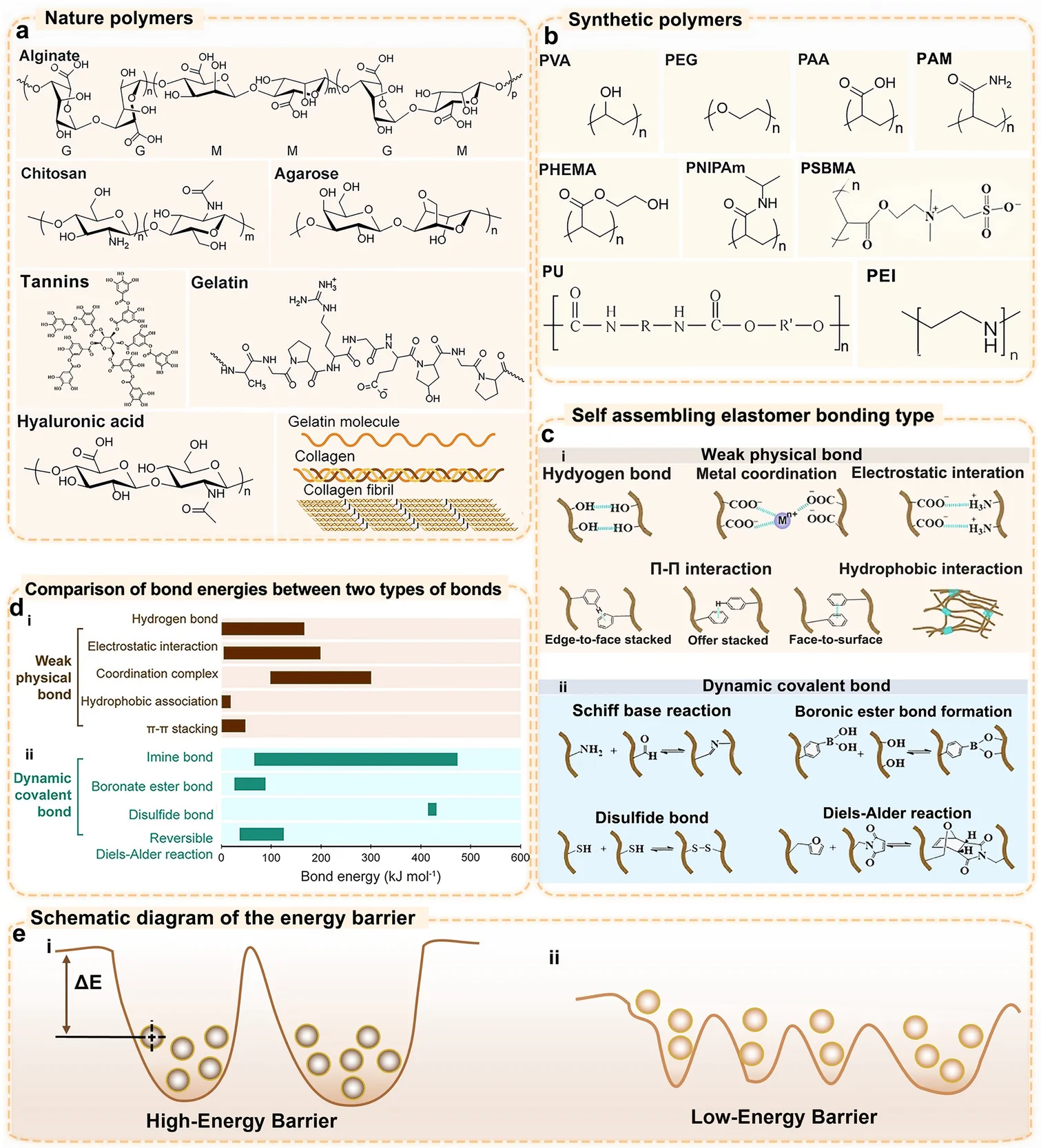
Dynamic hydrogen bond/covalent bond-driven cellulose elastomer self-assembly types. Reproduced with permission from Ref. [95, 107], Copyright 2024 Springer Nature and, Copyright 2021 American Chemical Society. **a** Natural polymer species (including alginate, chitosan, hyaluronic acid, agarose, gelatin (molecule, protein, fiber)). **b** Synthetic polymer species (including polyvinyl alcohol, polyethylene glycol, polyacrylic acid, polyacrylamide, polyhydroxyethyl methacrylate, poly(N-isopropylacrylamide), polysulfobetaine methacrylamide, polyurethane, polyethyleneimine). **c** Characteristics of self-assembly-driven molecular bonding types, including weak physical bonding interactions (hydrogen bonding, metal coordination bonding, electrostatic interactions, π-π interactions, hydrophobic interactions (edge-to-face stacking, offset stacking, and face-to-face stacking), and dynamic covalent bonds (Schiff base reaction, boronate ester bond, disulfide bond, Diels–Alder reaction)). **d** Comparison of weak bond and covalent bond energies. **e** Energy barrier comparison diagram. i. Traditional covalent crosslinking network (enthalpy-dominated, low entropy); ii. Dynamic bond-exchange network (entropy-enthalpy synergistic)

According to existing research studies, cellulose elastomers are typically not composed solely of cellulose [15, 54]. To meet the demands of energy harvesting and self-powered sensing, they often require interaction with natural or synthetic polymers to achieve structural and functional diversity, thereby fulfilling specific requirements such as elasticity, toughness, and environmental adaptability. Common natural polymers include alginates, chitosan, and hyaluronic acid, while synthetic polymers encompass polyvinyl alcohol, polyethylene glycol, polyacrylic acid, and polyacrylamide (Fig. 4a, b) [95].

Cellulose self-assembles with these polymers through noncovalent interactions-hydrogen bonds, van der Waals forces, electrostatic interactions, and hydrophobic effects-to form ordered structures and functional materials (Fig. 4ci) [96, 97]. However, they differ in their entropy regulation mechanisms. Taking the cellulose-PVA and cellulose-PEG systems as examples, the former achieves enthalpy-dominated self-assembly through a high-strength hydrogen bond network, significantly suppressing molecular chain conformational entropy to form low-entropy ordered structures [98, 99]. The latter, however, disrupts the cellulose-water structure through hydration to release translational entropy of water molecules while enhancing segmental mobility to increase conformational entropy, thereby achieving a dynamically stable system dominated by entropy increase [100, 101]. Furthermore, introducing natural polymers containing dynamic bonds (e.g., borate bonds, disulfide bonds, or metal coordination bonds) into the system provides an effective approach for constructing entropy-enthalpy synergistically regulated smart networks (Fig. 4cii) [102, 103].

These dynamic bonds undergo reversible bonding and network restructuring in response to external stimuli such as pH, light, or temperature. By increasing conformational entropy and accessible microstates, they not only significantly enhance the material’s self-healing capability, mechanical properties, and environmental adaptability but also enable intelligent response and dynamic regulation of macroscopic properties [95, 104, 105]. An energy landscape model explains the entropy-enthalpy synergistic regulation mechanism [106]. In traditional covalent networks, the system is permanently locked in a single stable state. Once pushed out of the energy well by external forces (e.g., deformation), it cannot return due to extremely high energy barriers, leading to permanent deformation or destruction, with configuration entropy frozen at a low level. In dynamic networks, reversible dynamic bonds (contributing enthalpy, ΔH) construct an energy landscape composed of multiple shallow energy wells (closely related to the bond energies of each dynamic bond in Fig. 4d). The energy barriers between these wells correspond to the activation energy for bond exchange. Under external stimuli (e.g., heat), the system gains energy to overcome these barriers, enabling exploration between different network configurations. This accessibility among numerous metastable states manifests as a significant increase in the system’s configurational entropy (−TΔS). Macroscopic adaptability—such as stress relaxation or shape reconfiguration—is the external expression of the system evolving from one high-entropy state to another via dynamic bond exchange during relaxation. Thus, dynamic bond switching acts as the molecular engine, configurational entropy serves as the driving force, and macroscopic adaptation emerges as the final behavior (Fig. 4e).

Therefore, entropy-driven self-assembly of cellulose and polymeric materials is crucial for constructing multifunctional cellulose-based elastomer structures, offering a promising pathway for their application in mechanical energy harvesting and flexible wearable electronics.

## Entropy-Driven Regulation of Self-Assembled Structural Performance in Cellulosic Elastomer

Self-assembly processes and Bouligand structures are often regarded as closely related in cellulose-based materials, representing common structural forms of self-assembly [108, 109]. However, recent studies indicate that entropy-driven self-assembly of cellulosic elastomers can yield macroscopic materials with highly diverse structural morphologies [110–115]. The final structure depends on the synergistic interaction of multiple factors, extending far beyond Bouligand configurations. To systematically understand the design principles of self-assembled structures and their structure–property relationships, this section will focus on reviewing the formation conditions and functional characteristics of several typical structures, including Bouligand structures, hierarchical layered structures, gradient structures, and functional interface structures. The analysis will cover their performance in dielectric properties, mechanical properties, and other aspects, aiming to provide theoretical foundations and experimental guidance for the targeted structural design of cellulosic elastomers.

### Structure of Self-Assembled Cellulosic Elastomer

The structural diversity of self-assembled ordered cellulosic elastomers is not without discernible patterns. Entropy serves as the “switch” that regulates the design of material conformation structures in this process. This study focuses on analyzing the formation conditions of Bouligand order, multilayer/multi-level network order, and gradient order, elucidating the structural evolution patterns in self-assembly processes from an entropy perspective.

#### Bouligand Structure

**Fig. 5:**
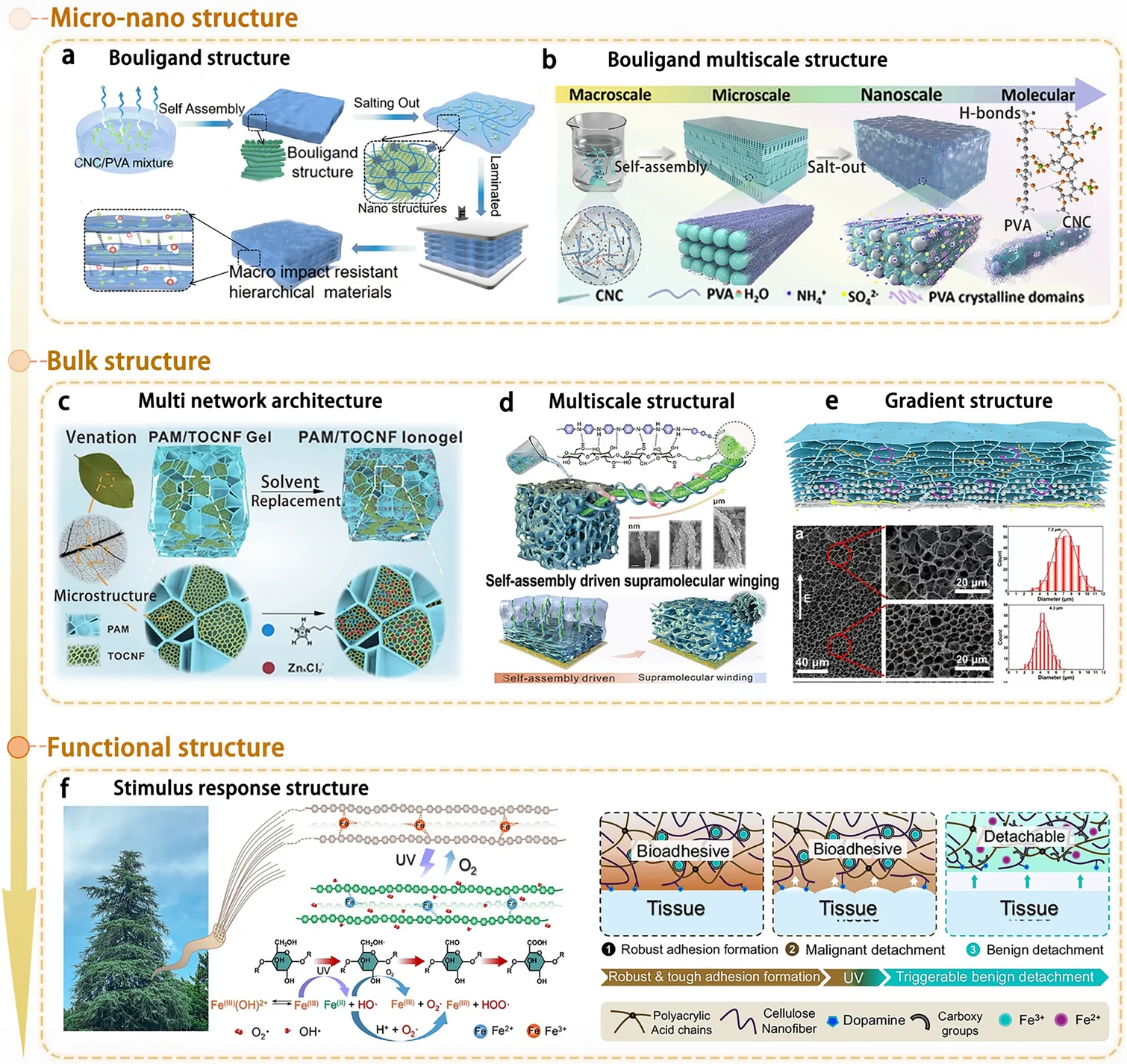
Structure regulation of self-assembly-driven cellulose elastomer. **a** and **b** represent the self-assembly driven cellulose elastomer Bouligand structure and multiscale micro-nano Bouligand structure, respectively. Reproduced with permission from Ref. [116, 117], Copyright 2024 American Chemical Society and Copyright 2024 American Chemical Society. **c**–**e** represent the macroscopic structure of self-assembly driven cellulose elastomer (multi-network structure, multiscale structure, gradient structure), respectively. Reproduced with permission from Ref. [114, 115, 118], Copyright 2024 John Wiley and Sons. **f** Stimuli-responsive structure of self-assembly-driven cellulose elastomer. Reproduced with permission from Ref. [110], Copyright 2024 Nature Publishing Group

The Bouligand structure found in nature (such as the exoskeletons of crustaceans) is a classic example formed by nanocellulose through multiscale self-assembly [116]. Although nanocellulose crystal suspensions can spontaneously assemble into Bouligand structures via electrostatic interactions and hydrogen bonding during solvent evaporation, their mechanical properties often fail to meet the requirements for elastomer applications. To address this, researchers dispersed cellulose nanocrystals in polyvinyl alcohol (PVA) solutions and introduced high-concentration ions during the self-assembly of PVA/CNC blends via salting-out treatment. This approach successfully constructed CNC/PVA composite elastomers with helical ordered structures (Fig. 5a, b) [117]. The initial dispersed state of this strategy is jointly maintained by the electrostatic repulsion (enthalpy) and translational entropy (entropy) of CNC, alongside the hydration (enthalpy) and conformational entropy (entropy) of PVA. Salting-out treatment triggers phase transition by shielding electrostatic forces (enthalpy regulation) and releasing translational entropy of water molecules (entropy-driven). During self-assembly, CNC autonomously arranges into a chiral liquid crystal template via entropy-driven organization, while PVA achieves structural stabilization by forming a hydrogen bond network (enthalpy-driven). This culminates in a synergistic assembly mode where “entropy drives structure formation, and enthalpy stabilizes the structure.”

#### Multiscale Hierarchical Network Structure

Similar to the formation of Bouligand structures. Researchers replaced water in the gel with the [BMIm]ZnxCly ionic liquid (composed of a 1-butyl-3-methylimidazolium cation and a zinc chloride anion cluster) via a displacement method [115]. This process first substantially increased the translational entropy of the system through solvent exchange (entropy-driven), while simultaneously forming strong coordination bonds between the ZnxCly anions in the ionic liquid and the hydroxyl groups on the TOCNF cellulose chains (enthalpy-driven). Subsequently, during the entanglement assembly between the PAM covalent network and TOCNF aggregates, TOCNF autonomously arranges into nanoscale pseudo-pore structures via entropy-driven self-assembly (nanoscale poly-TOCNF networks embedded within microscale PAM scaffold networks, mimicking leaf hierarchical grid structures), while the PAM network constructs microscale scaffolds through covalent crosslinking (enthalpy-driven). In the resulting hierarchical grid structure, supramolecular interactions (e.g., hydrogen bonds, ionic coordination) act as synergistic regulatory units, stabilizing the entropy-driven ordered assembly via enthalpy-driven mechanisms. This achieves a synergistic regulation mechanism where “entropy drives multi-level topological construction while enthalpy locks the network structure” (Fig. 5c).

Similarly, during the freeze-drying process of the CNF/PANI supramolecular system, the “disadvantageous” self-acceleration effect (The self-acceleration effect has been regarded as “undesirable” in the process of supramolecular autocatalytic polymerization) driven by entropy paradoxically forces the formation of multiple hydrogen bonds between polymer chains (enthalpy-driven) by restricting molecular motion degrees of freedom (reducing configurational entropy) [114]. These rapidly matched double hydrogen bonds act as molecular-level catalysts, compensating for entropy loss through exothermic reactions (enthalpy-driven) while guiding the multiscale entanglement of nanofibrillated cellulose and polyaniline via directed alignment (Fig. 5d). Ultimately, during the densification stage of the CNF substrate layer, the system undergoes macroscopic deformation driven by entropy increase (solvent molecular translational entropy rise) from solvent evaporation, while a dense hydrogen bond network (enthalpy-driven) locks the leaf-like elastomer structure (The dense CNF-supported substrate layer (leaf) is gently pressed into shape during the freeze-drying process of the CNF/PANI supramolecular self-assembled aerogel). This achieves synergistic self-assembly characterized by “entropy-driven molecular orientation induction and enthalpy-driven multi-level structural consolidation.”

#### Gradient Structure

Unlike the structures described above, the design of biomimetic gradient structures demands more stringent preparation conditions. Researchers successfully synthesized a multidimensional gradient porous conductive carbon nanofiber cellulose (CNC) elastomer material (Fig. 5e) composed of CNC and highly oriented nickel chains through self-assembly, utilizing processes such as freeze-drying combined with annealing [118, 119]. Analysis of the process entropy evolution revealed that vacuum drying provided a low-entropy environment, restricting molecular motion within Ni/CNC. Rapid cooling froze the ice-like Ni/CNC, gradually promoting its ordering. This significantly reduced collision-induced energy transfer and disorder, lowering the system’s entropy. Consequently, the frozen ordered structure was preserved and stabilized.

#### Stimulus–Response Function Interface Structure

The principle of stimulus–response function interface structure primarily relies on the ability of materials or systems to perceive and respond to specific external stimuli, achieving interfacial regulation by altering their own physical and chemical properties through self-assembly [120]. Cellulosic elastomers also serve as a typical example in the design of stimulus-responsive interfacial structures. The researchers utilized dopamine-modified CNF (CNF-DA) and polyacrylic acid (PAA) to co-assemble a supramolecular CNF-DA/PAA@Fe^3+^ hydrogel elastomer (Fig. 5f) that exhibits both reversible tough adhesion and easy photopeeling [110]. The UV light and oxygen induce a Fenton-like reaction, controlling the valence state of Fe ions within the gel. This dramatically alters the interfacial adhesion of the hydrogel elastomer, enabling excellent dynamic skin adsorption/desorption transitions. Metal ion-induced coordination and dynamic hydrogen bonding assemble the gel into denser aggregates—an entropy-reducing process—conferring superior structural stability. Under UV irradiation, the entire elastomer system undergoes stability disruption as Fe ion coordination weakens, representing an entropy-increasing process. Within the larger natural environment, the gel undergoes oxidative degradation by oxygen, reverting to a low-entropy stable state. Similarly, the research team utilized CNC co-assembled with 3-dimethyl(methacryloyloxyethyl)propane sulfonic acid ammonium salt (DMAPS) and methacrylic acid (MAA) to achieve an ordered orientation arrangement of P(DMAPS-MAA) hydrogel elastomers under shear stress [121]. Influenced by extensive dynamic hydrogen bonds, this hydrogel exhibits outstanding self-healing properties. More notably, the shape bending of the gel elastomer can be controlled by regulating pH and moisture content. During this process, high pH provides high ionic strength, causing the gel elastomer to disperse and exist in a high-entropy state. When pH decreases, hydrogen bonds drive the gel to re-aggregate and stabilize, shifting to a low-entropy state, thereby achieving dynamic shape tunability of the elastomer.

Similar stimulus-responsive interfacial structures can be activated not only by light and pH but also by heat, electric fields, magnetic fields, and other stimuli. These stimuli provide additional high-entropy conditions for the elastomer. It is precisely through the equilibrium between the stimulus source and cellulose self-assembly that dynamic interfacial changes are achieved.

#### Design Principles and Performance Trade-off Analysis of Self-Assembled Structures

Although the self-assembled structures presented in this section—such as Bouligand, layered, and gradient structures—exhibit rich morphological diversity, their formation processes all adhere to a common core principle: the final macroscopic structure arises from the competition and equilibrium between entropy and enthalpy [122]. Successful self-assembly is not merely an entropic increase process. Instead, it involves introducing specific enthalpy contributions (such as templates, external fields, or interfacial interactions) to guide the entropy maximization process toward predefined long-range ordered structures. This principle generates distinct structure–property trade-offs across different architectures, significantly influencing their mechanical and functional characteristics (e.g., dielectric properties). Specifically, Bouligand structures sacrifice some in-plane strength for exceptional toughness and impact resistance through helical arrangements. Their multi-level interfacial architecture simultaneously aids polarization and charge dissipation, optimizing dielectric loss [123]; Layered structures achieve outstanding in-plane strength and directional functional transport, though their interfacial layers may become mechanical weak points. Nevertheless, this highly anisotropic structure provides an ideal template for fabricating layered composites with high-dielectric constants and low losses through effective control of interfacial polarization [124, 125]. Gradient structures achieve smooth transitions in mechanical properties and effective mitigation of stress concentration through continuous variations in composition or porosity [126]. Their gradually changing dielectric constant distribution further positions them as unique platforms for high charge transport and gradient dielectric materials [127]. Thus, precise control over entropy/enthalpy equilibrium and a deep understanding of the inherent “mechanical-functional” trade-offs within specific structures are key to the targeted design and optimization of these multifunctional materials.

### Properties of Self-Assembled Cellulosic Elastomer

Entropy-driven self-assembly provides a powerful mechanism for tailoring the properties of cellulose-based elastomers through dynamic bonding and hierarchical structural organization. This approach enhances mechanical strength and toughness via intermolecular interactions such as hydrogen bonding, enables self-healing capabilities through reversible dynamic covalent and noncovalent bonds, and improves dielectric performance by promoting structural ordering and polarization modulation. These strategies collectively offer a versatile pathway for engineering multifunctional cellulosic elastomers with programmable properties.

#### Modulating Mechanical Performance

**Fig. 6:**
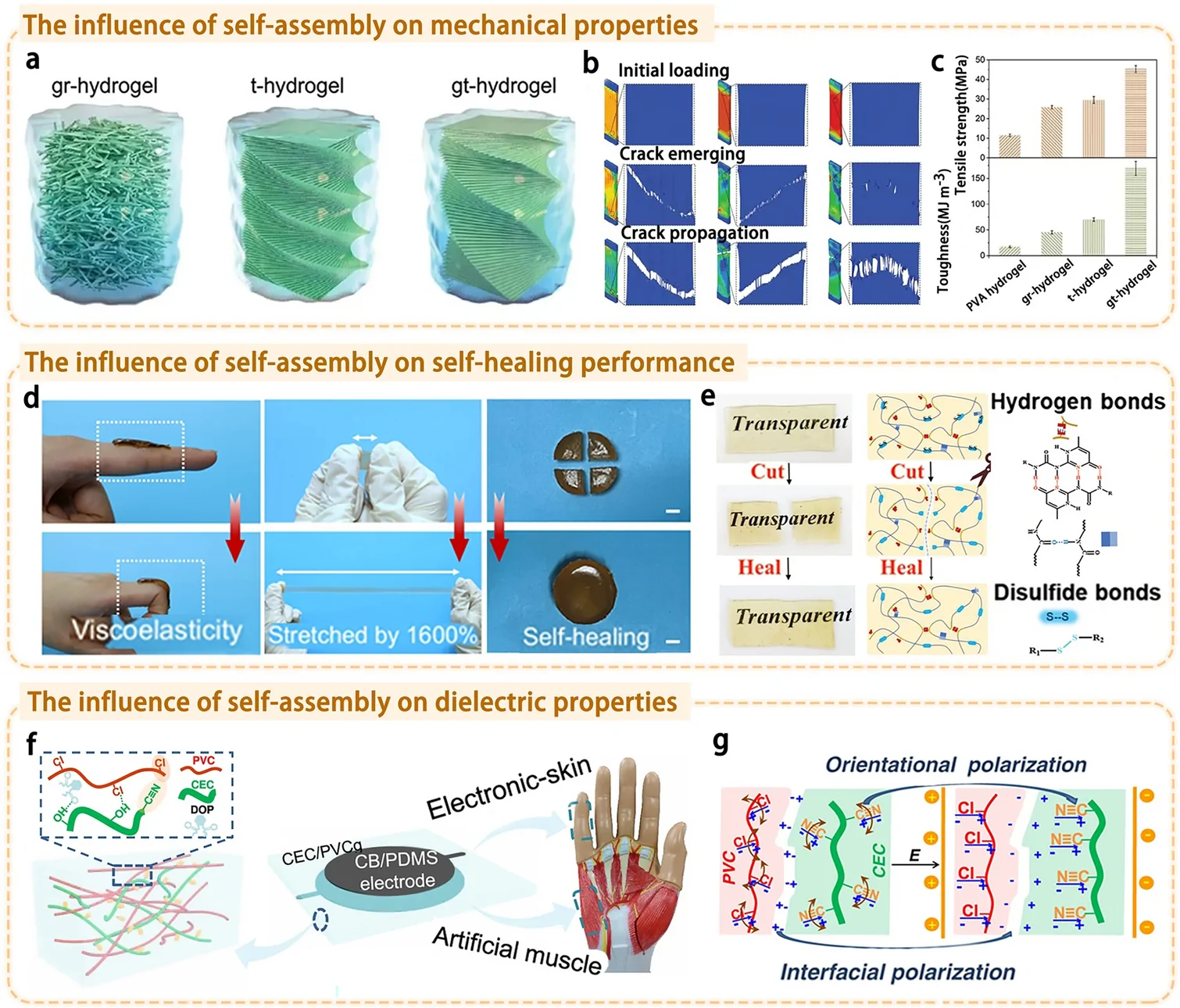
Entropy-driven self-assembly regulates the performance of cellulosic elastomers. **a**–**c** Mechanism of self-assembled structure of cellulose elastomer enhancing the mechanical properties of cellulose elastomer. Reproduced with permission from Ref. [128], Copyright 2024 John Wiley and Sons. **d** and **e** Self-assembly-driven regulation of self-healing properties of cellulosic elastomers. Reproduced with permission from Ref. [110, 130], Copyright 2023 John Wiley and Sons and Copyright 2024 Nature Publishing Group. **f** and **g** Self-assembly-driven regulation of dielectric properties of cellulosic elastomers. Reproduced with permission from Ref. [131], Copyright 2023 Nature Publishing Group

Entropy-driven cellulose self-assembly forms ordered structures through intermolecular interactions such as hydrogen bonds and van der Waals forces. This enables materials to disperse stress more effectively under external forces, significantly enhancing their strength and toughness (Fig. 6a–c) [128]. This process can also construct supramolecular systems (such as strong and tough cellulose supramolecular hydrogels) or co-assemble with nanoparticles, polymers, and other components to form composite materials (such as cellulose-bentonite hydrogels, which exhibit both high strength and low-temperature resistance), further enhancing mechanical properties [129]. In general, cellulose self-assembly offers a promising avenue for optimizing the mechanical properties of materials by controlling self-assembly conditions and incorporating other components, leading to broad application prospects.

The classification of mechanical properties for entropy-driven self-assembled cellulosic elastomers is shown in Table 2. A summary of recent comparisons of the mechanical properties (tensile strength, fracture strain, and elastic modulus) of entropy-driven cellulose elastomers is provided to highlight the performance differences between cellulose and various polymer assemblies. Based on the principle of entropy-driven self-assembly, a flexible, multifunctional, wearable bacterial cellulose@Fe_3_O_4_/carbon nanotube/Ti_3_C_2_T_x_ composite film with an asymmetric gradient structure was prepared using a hydrogen-bonding self-assembly strategy. The asymmetric gradient multilayer structure minimizes nanofiller agglomeration and maximizes interlayer hydrogen-bonding interactions, endowing the composite film with excellent mechanical properties [132].

**Table 2:** Comparison of mechanical properties of cellulosic elastomers

Elastomeric Materials	Tensile strength (MPa)	Fracture strain (%)	Elastic modulus (MPa)	References
PVA/nanocellulose	15.0	12.0	60.0	[133]
PVA/BC	36.2	47.0	-	[134]
Deacetylated cellulose acetate/polyurethane nanofiber	21.0	91.1	23.5	[135]
a-CNFs@PVA	39.0	4.5	1100.0	[136]
PolyC/CNF	28.0	1.7	1800.0	[137]
CNC/PVA@UiO-66-(COOH)_2_	1.7	55.0	-	[138]
Flexible photonic film (FPFS)	15.7	5.8	901.0	[139]
ICN	16.0	38.4	190.0	[140]
Ammonium sulfate-treated PAM/MC	4.4	690.0	3.8	[141]
D1C1-1	-	24.3	7.4×10^–3^	[142]
Reinforced cellulose-protein (RCP)	30.7	160.0	64.1	[143]
Cel-BF_4_	3.4	-	24.0	[144]
PAA-g-QCE/PVA	1.1	465.0	0.3	[145]
CNF/PU hybrid	0.25	23.0	-	[146]
CNF-reinforced silica	0.08	45.0	1.9	[147]

Furthermore, using bio-based gelatin/glycerol (GG) elastomer as the dielectric elastomer matrix, CNC with abundant hydroxyl groups disrupts hydrogen bonds between gelatin molecules under entropy-driven self-assembly and forms stronger hydrogen bonds with them. The favorable interfacial interactions between GG and CNC, along with the excellent dispersion of CNC within the GG matrix, confirm the pivotal role of entropy-driven assembly hydrogen bond formation in enhancing mechanical properties [148].

Unlike organic polymers, which readily stabilize each other through intermolecular hydrogen-bonding interactions, the uniform dispersion of inorganic nanoparticles within nanocellulose films requires careful regulation of entropy-driven thermodynamic parameters (temperature, particle concentration, etc.) [149], through in situ layer-by-layer self-assembly, structurally uniform bacterial cellulose/graphene oxide (BC/GO) hydrogel elastomers. Strong hydrogen bonds between BC and GO ensure tight bonding between one-dimensional and two-dimensional components, while the layer-by-layer cultivation mode improves GO nanosheet dispersion within the BC matrix. This promotes mechanical binding between BC nanofibers and GO nanosheets, forming a network structure. Strong hydrogen bonds, tight mechanical entanglement, and uniform distribution collectively enhance the mechanical properties of BC/GO hydrogels [150].

When preparing nanocomposites, the uniform dispersion of nanoparticles in nanocellulose films is critical. Therefore, the layer-by-layer self-assembly technique is an effective method to construct materials with high mechanical strength and uniformity. Thick bacterial cellulose/graphene oxide (BC/GO) hydrogels with a uniform structure were prepared by layer-by-layer assembly in situ. The strong hydrogen bonds formed between BC and GO ensured the tight binding of 1D and 2D components, while the layer-by-layer culture mode improved the dispersion of GO nanosheets in the BC matrix and promoted the mechanical bundling of BC nanofibers to GO nanosheets, forming a vein-like structure. Strong hydrogen bonds, tight mechanical bundling, and uniform distribution together enhanced the mechanical properties of the BC/GO hydrogels. Exceptional mechanical properties—high strength and toughness—ensure that devices resist fatigue damage, crack propagation, or permanent failure under repeated mechanical deformation such as bending, stretching, and compression. This is crucial for wearable devices and electronic skins requiring long-term, stable operation [151, 152].

#### Self-Healing Performance

The performance regulation of self-healing materials relies on the reversible breaking and recombination of dynamic bonds, and entropy-driven self-assembly plays an important role in this process by maximizing the system disorder (entropy increase). Dynamic bonds spontaneously recombine at the damage interface to achieve a balance between energy dissipation and structural recovery [153]. In recent years, multi-network designs based on dynamic covalent bonds (such as Schiff base bonds and disulfide bonds) and noncovalent bonds (such as hydrogen bonds, metal coordination, and π-π stacking), combined with entropy increase-dominated spontaneous recombination mechanisms, have become a key strategy for improving the self-healing efficiency of materials [154]. Notably, different self-healing mechanisms exhibit significant variations in their entropy requirements and thermodynamic constraints.

Self-healing systems dominated by dynamic covalent bonds require substantial entropy increase to overcome bond-breaking energy barriers: temperature-sensitive dynamic disulfide bonds synergizing with hydrogen bonds enable efficient reconstruction of covalent-noncovalent crosslinked networks in cellulose materials, while the material’s strong temperature dependence in viscoelasticity confers exceptional reprocessing and reshaping capabilities [155]. In cellulose bioplastics containing dynamic imine bonds, temperature-induced entropy increase activates imine bond-exchange reactions, enhancing molecular chain mobility and bond-exchange rates to drive crosslinking network reconstruction [156]. The dynamic reversibility of Schiff base bonds synergizes with the energy dissipation properties of metal coordination bonds to form an entropy-driven effect, conferring highly efficient self-healing capabilities upon hydrogels (Fig. 6d) [157]. Flexible photonic films (FPFS) achieve self-healing through dual entropy-driven mechanisms: dynamic disulfide bond exchange and the chiral nematic structure of CNC (Fig. 6e) [139]. The covalent bond exchange requires significantly higher entropy increase than noncovalent interactions.

Noncovalent-dominated self-healing systems demand lower entropy increase but are constrained by molecular motion degrees of freedom: in aqueous polyurethane—cellulose nanofibre (SWPU-CNF) elastomers exhibit self-healing through hydrogen bond disruption by entropic increase upon heating, followed by hydrogen bond reformation upon cooling. The CNF induces a looser hard domain structure in the composite, enhancing molecular mobility and reducing dynamic bond activation energy, thereby further optimizing self-healing efficiency [158]; Microfibrillated cellulose (MFC)-reinforced PVA-borax hydrogels exhibit outstanding self-healing capability and mechanical strength, alongside pH-responsive sol–gel reversible transitions. Their self-repair and dynamic reversibility stem from flexible polymer chains, hydrogen bond reconstruction, and reversible diol borate bonds [159]. Thermoresponsive supramolecular hydrogels achieve rapid gel-sol transitions under thermal stimulation-induced entropy increase through hydrogen bonding and π-π stacking interactions [160].

The introduction of self-healing capabilities has significantly enhanced the practical value and reliability of cellulose elastomer devices in mechanical energy harvesting and self-powered sensing applications [161, 162]. Specifically, this property first ensures the recoverability of device functionality: when accidental damage causes circuit breaks or electromechanical failure, the material can achieve structural healing and functional regeneration through dynamically reversible chemical bond rearrangement. Furthermore, it endows devices with the potential to withstand complex, unpredictable mechanical stresses. This is crucial for equipment requiring long-term stable operation in dynamic environments—such as electronic skin and implantable monitoring systems—laying the material foundation for constructing highly robust intelligent systems.

#### Dielectric Property

The optimization of dielectric properties relies on the balance between polarization mechanisms and energy loss within the material [163], and entropy-driven self-assembly can significantly affect the dielectric response by regulating the dynamic arrangement of molecules or nanostructures (such as hydrogen bond networks and dipole orientations) [164]. The entropy-driven self-assembly process maximizes the disorder of the system (entropy increase), prompting the material to form a stable structure with the lowest energy in dynamic equilibrium, thereby optimizing the synergistic relationship between the dielectric constant (ε) and the dielectric loss (tanδ) [165, 166]. Specifically, entropy-driven self-assembly can both reduce free polar groups by enhancing hydrogen-bonding interactions between components, thereby regulating polarization relaxation to influence the dielectric constant [167]; and promote the formation of interfaces and hydrogen bonds with larger dipole moments, thus enhancing the dielectric constant of composite materials [168]. A summary of recent studies on entropy-driven dielectric properties (dielectric constant, dielectric loss tangent) in cellulose elastomers is presented to highlight the contrast between cellulose and various polymer-assembled dielectric materials (Table 3).

**Table 3:** Comparison of dielectric properties of cellulosic elastomers

Elastomer Material	Frequency (Hz)	Dielectric Constant	Dielectric Loss	References
Regenerated Cellulose	10^3^	13.0	0.030	[45]
RC/AONS/PVDF Ternary	10^3^	10.2	0.021	[175]
RC/PVDF Composite	10^3^	9.0	0.030	[176]
CA/PMMA Composite	10^3^	6.7	0.028	[177]
CNF/PVA	10^−1^	4.6 × 10^9^	2.020	[178]
CMC	-	78.1	-	[179]
CNF/CNT	10^10^	2.0	0.500	[180]
GNS/Cellulose	10^10^	7.2	1.860	[181]
CNT/Cellulose-Derived	10^10^	7.5	1.150	[182]
PPy/CA	10^10^	3.3	0.360	[183]

Ultralight aerogels with aligned pores were synthesized from CNC and agarose (AG) through techniques such as ice-crystal-induced alignment, freeze-drying, and chemical modification. The oriented structure (including CNC arrays and aligned pores) and heterojunction with electron transfer pathways of the CNC/AG aerogels reduced the dielectric loss, exhibiting a synergistic effect in improving the electromechanical conversion efficiency and triboelectric performance [169].

Electric field-induced molecular self-assembly alignment also demonstrates significant regulatory effects. By studying the alignment direction of sodium carboxymethyl cellulose microfibers in silicone elastomer (PDMS) under a DC electric field, composite films with high-dielectric constants can be produced. Compared to composite films without CMC alignment, the aligned composite films exhibit a significant increase in dielectric constant. This is because their chain-like structure resembles the parallel model of two-phase composites, which exhibit higher dielectric constants when the second phase is aligned parallel to the electric field, thereby enhancing the dielectric properties [170]. Furthermore, self-assembly-driven chemical crosslinking regulates the mobility of molecular chains. For example, in the preparation of epichlorohydrin (ECH)-crosslinked regenerated cellulose membranes (RCCE), the reaction between cellulose hydroxyl groups and ECH weakens the hydrogen bond network and reduces crystallinity, releasing more freely mobile -OH groups, thus increasing the dielectric constant [171].

Functionalization of cellulose with cyanoethyl groups, followed by introduction into plasticized PVC, yields CEC/PVC elastomers. The large orientational polarization of the C≡N dipole moment expands the interfacial capacitance, endowing the elastomer with a high-dielectric constant [131] (Fig. 6f, g). Unlike the aforementioned-mechanism that merely increases the dielectric constant, achieving synergistic high-dielectric constant and low dielectric loss is key to enhancing the high electrical output and long service life of cellulose elastomer materials as flexible electronic components [172, 173]. Flexible regenerated cellulose/polypyridine (RC-PPy) conductive composite films achieve synergistic high-dielectric constant and low dielectric loss values by forming continuous conductive networks. Their loss factor (ε′′) comprises three distinct effects:

2 $$ \varepsilon^{\prime\prime} = \varepsilon^{\prime\prime}_{dc} + \varepsilon^{\prime\prime}_{MW} + \varepsilon^{\prime\prime}_{D} $$

Where ε′′_*dc*_, ε′′_*MW*_, and ε′′_*D*_ represent DC conductivity, interfacial polarization, and dipole orientation, or the Debye loss factor, respectively. The total frequency-dependent conductivity of the composite can be expressed as:

3 $${\sigma }{\prime}={\sigma }_{dc}+{\sigma }_{ac}$$

where ${\sigma }_{dc}$ and ${\sigma }_{ac}$ are the DC and AC conductivities, respectively [174].

The dielectric properties enhanced by entropy-driven self-assembly strategies is crucial for device performance. In triboelectric nanogenerators, a higher dielectric property strengthens the confinement of triboelectric charges and electrostatic induction capabilities, directly increasing charge density and power output [184]. Simultaneously, when employed as the dielectric layer in capacitive sensors, its exceptional polarization properties convert minute pressures or strains into significant capacitance changes, enabling highly sensitive signal detection [185]. This dual functionality makes dielectrically optimized cellulose elastomers valuable for both efficient energy harvesting and precision sensing applications.

To clarify the design principles of self-assembled structures and correlate them with specific energy-harvesting mechanisms is crucial for achieving high-performance devices. This study thoroughly explores the structure–property relationships between various self-assembled structures—such as Bouligand structures, multiscale structures, gradient structures, and dynamically stimulus-responsive network structures—and their core properties (mechanical properties, dielectric properties, self-healing properties, and electromechanical conversion properties). It also compiles and organizes the contributions of these structural characteristics to the field of energy harvesting. Relevant findings are summarized in Table 4.

**Table 4:** Comparative analysis of common self-assembled structural properties in energy-harvesting applications

Structure	Mechanical Properties	Dielectric Properties	Self-healing capability	Electromechanical Conversion Performance	References
Bouligand		**–**	**–**		[117]
Bouligand		**–**	**–**		[186]
multiscale		**–**	**–**		[152]
multiscale		**–**	**–**		[187]
multiscale	**–**		**–**		[188]
Gradient		**–**	**–**		[189]
Gradient	**–**	**–**	**–**		[190]
Gradient		**–**	**–**	**–**	[191]
Dynamic Stimulus Response		**–**		**–**	[158]
Dynamic Stimulus Response		**–**			[192]
Dynamic Stimulus Response		**–**	**–**		[193]

## Cellulosic Elastomer for Electromechanical Conversion and Self-Powered Sensing

Entropy-driven self-assembly systematically optimizes the electromechanical response properties of cellulose elastomers by precisely regulating molecular orientation, hydrogen bond rearrangement, and multiscale structural formation. Regarding dielectric performance, molecular chain orientation induces more directional dipoles that respond more actively to applied charges, thereby enabling more efficient dipole polarization. Conversely, the ordered arrangement of cellulose chains reduces distances between crosslinking sites, hindering impurity ion transport and thereby suppressing dielectric relaxation of dipoles to a certain extent. [184, 194]. Hydrogen bond network restructuring directly regulates dielectric polarization strength and relaxation behavior by altering dipole density, orientation, and rotational barriers [195, 196]. Multiscale structures significantly enhance interfacial polarization by constructing interfaces and defects across different dimensions (from molecular to nano- and micrometers), thereby enabling control over material dielectric properties [197]. High-dielectric properties critically influence common mechanical-to-electrical energy conversion generators (piezoelectric, triboelectric, and dielectroelastic generators). As a key medium, dielectric properties profoundly affect the performance of various electromechanical energy conversion devices through distinct physical mechanisms: A high-dielectric constant enhances a material’s ability to store and maintain polarization charges induced under stress, thereby effectively increasing the apparent piezoelectric voltage coefficient under identical strain conditions [198]. In triboelectric power generation, high-dielectric constants significantly enhance open-circuit voltage and short-circuit current by amplifying dielectric polarization and electrostatic induction effects; for dielectroelastic generators, high-dielectric constants directly increase energy density, while low dielectric loss and high breakdown strength jointly ensure charging/discharging efficiency and upper operational field limits [199, 200].

Additionally, entropy-driven self-assembly endows materials with enhanced mechanical properties and self-healing capabilities. This significantly boosts the environmental adaptability, durability, and functional reliability of energy harvesting and sensing systems: optimized mechanical properties—such as high toughness and stretchability—ensure structural integrity and stable output during complex deformations, while directly improving piezoelectric/triboelectric energy conversion efficiency by effectively transmitting and amplifying external stresses. Self-healing capabilities autonomously repair microcracks and circuit breaks caused by mechanical fatigue or damage [201, 202]. This not only restores the device’s mechanical integrity to sustain long-term performance but, more critically, reconstructs disrupted conductive pathways and dielectric isolation layers. Consequently, electrical output signals (e.g., current, resistance) regain consistency, substantially extending sensor lifespan under harsh conditions and enhancing data acquisition reliability. Ultimately, this enables intelligent, robust, and sustainable self-powered sensing. These self-assembly-induced multiscale structural features synergistically enhance material mechanical durability and self-healing capabilities while establishing a systematic regulation pathway from molecular order to macroscopic performance. This section will delve into the regulatory mechanisms governing the dielectric, piezoelectric, and triboelectric properties of cellulose elastomers, providing theoretical foundations for developing high-performance mechanical energy harvesting and self-powered sensing materials.

### Piezoelectricity

Piezoelectric power generation in elastomers primarily relies on the piezoelectric effect to convert mechanical energy into electrical energy, with its performance determined by both the piezoelectric coefficient and strain rate. By optimizing the intrinsic properties of piezoelectric materials, designing energy-harvesting structures such as multilayer stacks and cantilever beams, and integrating impedance-matching circuits with energy management chips, output efficiency can be significantly enhanced. This provides critical technological support for low-frequency energy harvesting and self-powered sensing applications.

#### Principle of Piezoelectric Power Generation

Piezoelectric generators, as a type of self-powered generator, boast advantages such as simple fabrication, low cost, and high energy conversion efficiency, thus finding wide application in fields like low-frequency energy harvesting, wearable devices, and electronic equipment [203, 204].

A piezoelectric nanogenerator (PENG) comprises piezoelectric materials, metal electrodes, and an encapsulation casing. Among these, piezoelectric materials serve as the core component of PENG, responsible for converting mechanical energy into electrical energy [205]. Electrodes function to collect and transmit the electric charges generated by the piezoelectric materials. The encapsulation casing is typically made of lightweight yet durable materials, providing structural support for the PENG while effectively protecting the electrodes and piezoelectric materials from external hazards (e.g., dust, moisture, and physical damage) [206].

**Fig. 7:**
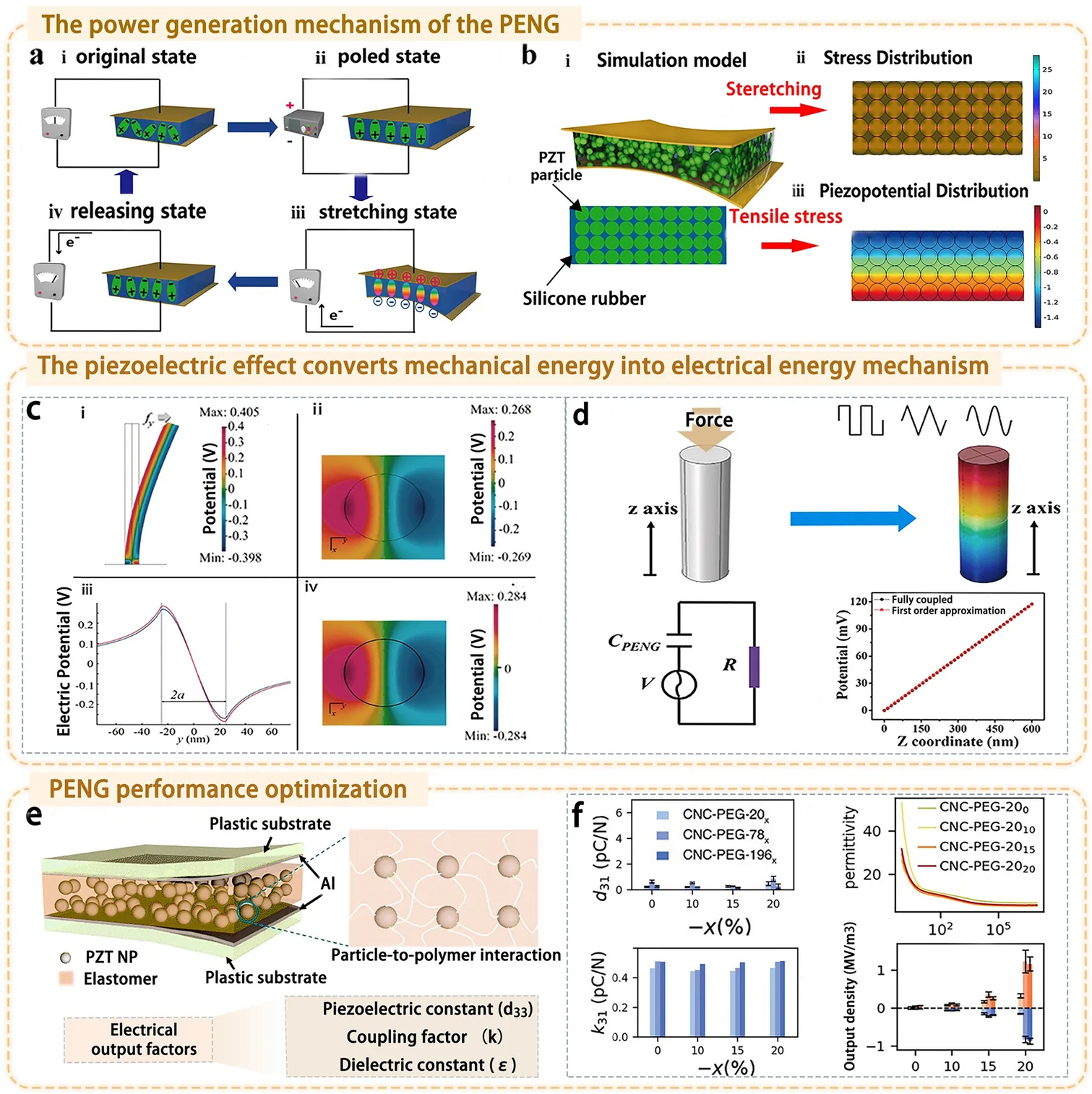
Piezoelectric elastomer generator mechanism. **a** PENG generator mechanism. **b** simulation model, stress, and piezoelectric potential distribution under tensile conditions. Reproduced with permission from Ref. [207], Copyright 2018 Elsevier. **c** (i) Piezoelectric potential distribution of deflected nanowires (length 600 nm, diameter 50 nm) under a lateral force of 80 nN. (ii) and (iii) piezoelectric potential of the PENG cross section (z0 = 300 nm) calculated analytically using the finite element method. (iv) comparison of piezoelectric potentials calculated by the two methods. Reproduced with permission from Ref. [208], Copyright 2007 American Chemical Society. **d** Equivalent circuit of the PENG, where the PENG is treated as a voltage source. Reproduced with permission from Ref. [209], Copyright 2021 Elsevier. **e** Energy-harvesting model of the PENG. Reproduced with permission from Ref. [210], Copyright 2018 Royal Society of Chemistry. **f** Major factors influencing the output performance of the PENG (piezoelectric constant, electromechanical coupling coefficient, dielectric constant) and their regulation of electrical output. Reproduced with permission from Ref. [21], Copyright 2025 John Wiley and Sons

The working principle of PENG lies in converting mechanical energy into electrical energy via the piezoelectric effect (Fig. 7a, b) [209]. The fundamental equations of the piezoelectric effect are as follows:

4 $$\left\{\begin{array}{c}{\sigma }_{p}={c}_{pq}{\varepsilon }_{p}-{e}_{kp}{E}_{k}\\ {D}_{i}={e}_{iq}{\varepsilon }_{p}+{\kappa }_{ik}{E}_{k}\end{array}\right.$$

where ${\sigma }_{p}$ denotes the stress tensor, ${D}_{i}$ the electric displacement,${c}_{pq}$ the elastic modulus tensor,${\varepsilon }_{p}$ the strain tensor, ${e}_{kp}$ the piezoelectric tensor, and ${E}_{k}$ the electric field. ${\kappa }_{ik } \rm and {e}_{iq}$ denote the dielectric tensor and piezoelectric tensor, respectively. Equation 4 is a coupled equation, for which an analytical solution often does not exist (Fig. 7ci). To tackle this issue, Gao et al. employed a perturbation expansion method to solve the coupled equations, deriving an expression for the piezoelectric potential distribution with high accuracy (with a deviation of less than 6%) (Fig. 7cii-civ) [208]:

5 $${D}_{Ri}={e}_{{i}_{P}}{\varepsilon }_{P}$$

6 $$\left\{\begin{array}{c}{\rho }^{R}=-\frac{\partial {D}_{Ri}}{\partial {x}_{i}}\\ {\Sigma }^{R}={n}_{1}\cdot {D}_{Ri}\end{array}\right.$$

In these equations, ${D}_{Ri}$ denotes the residual displacement, while ${\rho }^{R}$, ${\Sigma }^{R}$ denote the volume charge and surface charge, respectively. Thus, the mechanism of PENG can be described as follows. Under external force, the volume charges and surface charges generated by the piezoelectric material induce a potential drop across the PENG electrodes. Once PENG is connected to an external load, this potential drop drives electron flow, thereby generating electricity. As shown in Eq. 5, electric displacement is determined by the strain tensor and piezoelectric tensor. Based on Eq. 6, we assume that the piezoelectric material is uniformly distributed internally, with only residual surface charges present. The equivalent circuit of PENG can be modeled as a voltage source. ${V}_{0}$ in series with a capacitor ${C}_{P}$, thereby yielding the voltage source equation (Fig. 7d) as shown in Eq. 7:

7 $${V}_{0}=\frac{{e}_{iP}{\varepsilon }_{P}}{{C}_{P}}$$

where ${C}_{P}$ Denotes the capacitance of PENG. When PENG is connected to a capacitive load, the existence of ${C}_{P}$ Results in the output voltage being lower than the open-circuit voltage. Recently, Wang et al. identified a key parameter governing the mechanical-to-electrical energy conversion, the displacement current density (Eq. 8) [211, 212]:

8 $$J=\frac{\partial {D}_{R}}{\partial t}={e}_{ip}\frac{\partial {\varepsilon }_{P}}{\partial t}$$

In such cases, the equivalent circuit of PENG can be modeled as a current source in parallel with ${C}_{P}$. Equation 8 establishes a relationship between the strain rate, piezoelectric coefficient, and PENG output. From this relationship, it can be concluded that a higher strain rate and a superior piezoelectric coefficient will lead to a greater PENG output. Therefore, under fixed external load conditions. In addition to using soft, stretchable elastic materials as the substrate in piezoelectric elastomer generators. The design and development of piezoelectric materials with excellent piezoelectric coefficients and high strain rates are of great significance. This is crucial for the efficient conversion of mechanical energy to electrical energy in PENG.

#### Optimization of Piezoelectric Power Generation Performance of Elastomers

Optimizing piezoelectric output performance requires synergistic regulation across three dimensions: material properties, structural design, and energy management [213]. At the material level, piezoelectric constants (d_33_), dielectric constants, and electromechanical coupling coefficients are key performance-determining parameters (Fig. 7e, f) [21, 210, 214]. Intrinsic material performance can be significantly enhanced through compositional regulation, texturing, and composite modification. Taking the typical piezoelectric polymer PVDF as an example, its electromechanical conversion capability fundamentally relies on the formation and orientation of the electroactive β phase. From the perspective of the polarization mechanism, the generation of the piezoelectric effect necessitates the condition of ‘directional alignment of dipoles’. Currently, three primary strategies enhance dipole alignment to achieve superior piezoelectric performance: firstly, inducing dipole alignment in piezoelectric materials through the electrostatic polarization process; secondly, controlling material orientation (termed texturing); and thirdly, generating self-polarization effects by introducing interfacial polarization between different components Regarding structural optimization, multilayer stacking designs enhance low-stress response, while cantilever structures combined with mass tuning optimize resonant frequency matching. Flexible electrode designs ensure stable contact under large deformations [215–218]. The thin-walled characteristics of porous structures substantially reduce material Young’s modulus [219], enabling significant deformation under external forces while promoting internal dipole deflection to intensify piezoelectric effects [220]. Micro/nanostructures enhance piezoelectric performance through geometric strain confinement effects, high strain tolerance, and ordered dipole alignment [221–223].

In terms of structural optimization, multilayer stacked designs can enhance low-stress response, while cantilever beam structures combined with mass block tuning can optimize resonance frequency matching. Flexible electrode designs guarantee stable contact under large deformations. Optimizing the energy management system is crucial for practical applications. Precise impedance-matching circuit design enables maximum power transmission.

Integrated energy-harvesting chips improve AC-DC conversion efficiency. The combination with energy storage systems effectively addresses the issue of intermittent energy output. Current research remains challenged by key technical bottlenecks, such as wideband energy harvesting and efficient conversion under small stresses. These advancements will propel the application of piezoelectric technology in low-frequency vibration energy harvesting and self-powered sensing, providing reliable micro-energy solutions for the Internet of Things and intelligent systems.

### Elastomer Triboelectric Nanogenerator

Triboelectric nanogenerators (TENG) convert mechanical energy into electricity through contact electrification and electrostatic induction, with output performance governed by surface charge density and dielectric characteristics [224, 225]. Performance is enhanced by selecting triboelectric pairs with high electron affinity differences, engineering micro/nano-structured surfaces, and implementing multilayer architectures to maximize contact area. Further improvements in energy conversion efficiency and operational stability are achieved through synchronous charge extraction circuits and impedance-matching strategies, making TENG a foundational technology for flexible wearable electronics and self-powered systems.

#### Principle of Elastomer Triboelectric Nanogenerators

**Fig. 8:**
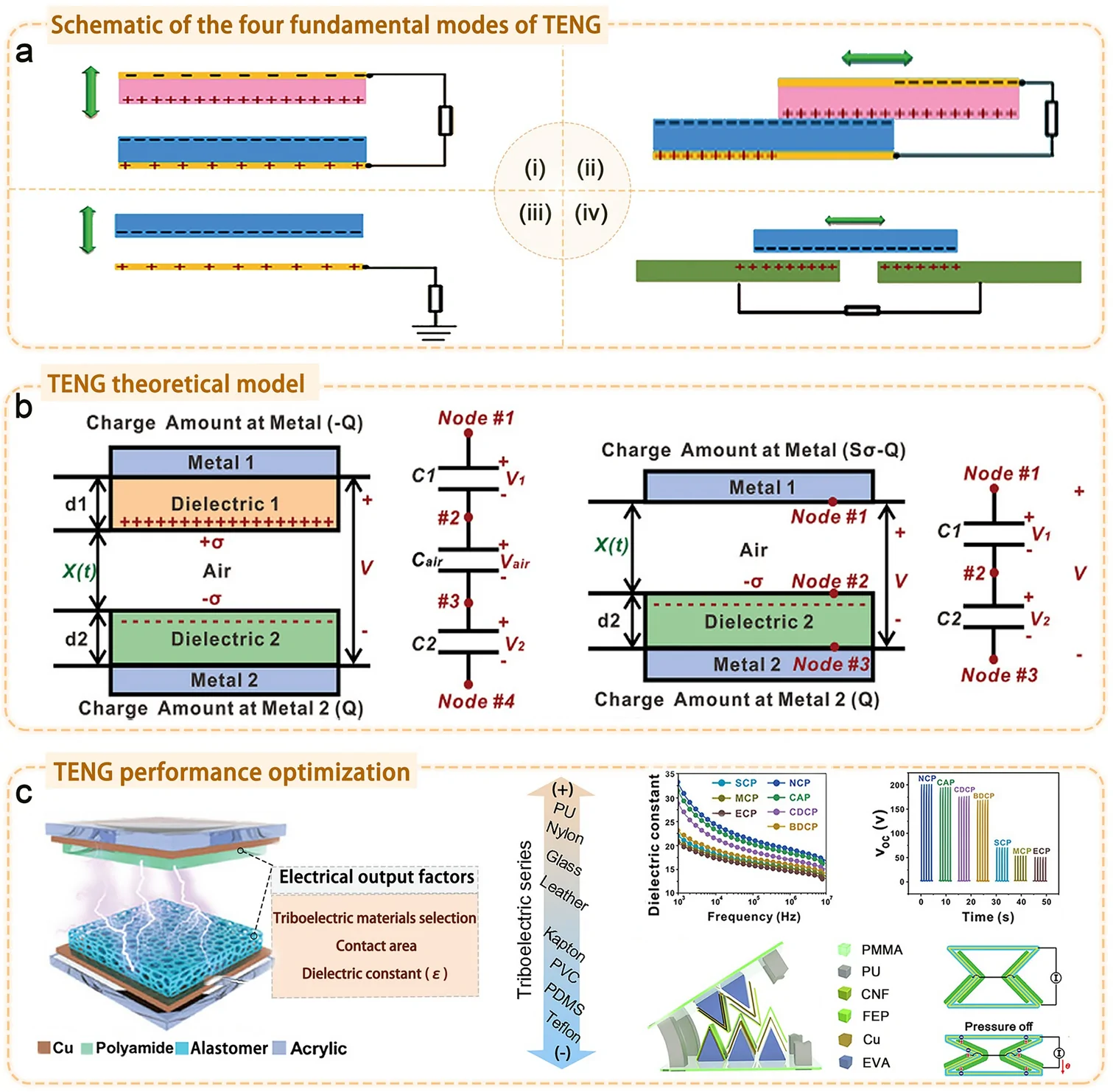
Schematic Illustration of TENG Working Mechanism. **a** Four basic modes of TENG and theoretical model diagrams. (ai) Vertical contact-separation mode. (aii) In-plane contact-sliding mode. (aiii) Single electrode mode. (aiv) Independent triboelectric-layer mode. Reproduced with permission from Ref. [246], Copyright 2014 Royal Society of Chemistry. **b** Theoretical model of dielectric-dielectric and conductor–dielectric TENG. Reproduced with permission from Ref. [233], Copyright 2015 Elsevier. **c** Electrical output regulation. Reproduced with permission from Ref. [241], Copyright 2025 American Chemical Society. Examples of factors influencing electrical output performance (triboelectric sequence, dielectric constant of the triboelectric layer, contact area). Reproduced with permission from Refs. [200, 237, 238], Copyright 2023 John Wiley and Sons and Copyright 2022 John Wiley and Sons, and Copyright 2023 Elsevier

TENG first introduced by Wang and his team in 2012, operates on the principles of electrostatic induction and the triboelectric effect [226–229]. Specifically, when two objects composed of distinct materials come into contact, disparities in their electron-confinement capabilities lead to the generation of equal and opposite charges at the contact interface. The charges formed on the material surfaces give rise to a potential difference in the external circuit. Electrons are propelled by this potential difference to move between the electrodes. If there is a load or a short-circuit in the external circuit, the charges will oscillate between the two electrodes. This process thus generates an electric current. TENG can be classified into four operational modes: vertical contact-separation mode, lateral sliding mode, single-electrode mode, and freestanding triboelectric-layer mode (Fig. 8a) [230–232]. One of the remarkable features of TENG is its ability to transform energy into electrical energy. This energy is almost any irregular and predominantly low-frequency type, sourced from human activities, machinery, and natural phenomena. Taking the typical vertical contact-separation mode as an example (Fig. 8b), the relationship between the induced voltage V transferred charge Q, and displacement x of the triboelectric layer is given by Eq. 9 [233, 234]:

9 $$V=-\frac{Q}{s{\varepsilon }_{0}}\left({d}_{0}+\text{x }\left(t\right)\right)+\frac{\sigma \text{x }\left(t\right)}{{\varepsilon }_{0}}$$

The open-circuit voltage is given by Eq. 10:

10 $${V}_{OC}=\frac{\sigma x \left(t\right)}{{\varepsilon }_{0}}$$

In the short-circuit (SC) state, V is 0. The transferred charge ${Q}_{SC}$ is given by Eq. 11:

11 $$ Q_{SC} = \frac{{S{ }\sigma {\text{x }}\left( t \right)}}{{d_{0} + x \left( t \right)}} $$

The short-circuit current (${I}_{SC}$) is given by Eq. 12:

12 $${I}_{SC}=\frac{\mathrm{d} {Q}_{sc}}{{\varepsilon }_{0}}=\frac{S \sigma {d}_{0}}{{{(d}_{0}+x \left(t\right))}^{2}} \frac{d\text{ x }}{d t }= \frac{S \sigma {d}_{0}v \left(t\right)}{{{(d}_{0}+x \left(t\right))}^{2}}$$

Therefore, the output performance of TENG directly depends on the surface charge density (σ) and effective dielectric thickness (${d}_{0}$) of the triboelectric material. Furthermore, in the parallel-plate capacitor model, σ is related to the capacitance (c) of the dielectric layer, and this relationship can be expressed by Eqs. 13 and 14 [235]:

13 $$\sigma =\frac{C V}{S}$$

14 $$C=\frac{S {\varepsilon }_{\gamma }{\varepsilon }_{0}}{d}$$

A higher dielectric constant and thinner dimensions are key to improving TENG output performance. Recent studies have shown that increasing the dielectric constant can effectively increase the total charge transfer density (${\sigma }{\prime}$) [236]. Its expression is given by Eq. 15:

15 $${\sigma }{\prime}= \frac{-{\sigma }_{0}{d}_{gap}}{{d}_{gap}+{d}_{0}/{\sigma }_{r}}$$

Herein, ${\sigma }_{0}$ denotes the equilibrium triboelectric charge density. ${d}_{gap}$ and ${d}_{0}$ represent the gap distance and dielectric film thickness, respectively, and ${\varepsilon }_{\gamma }$ is the dielectric constant. Under a fixed surface charge density, increasing the dielectric constant of triboelectric materials can effectively enhance TENG output performance [236]. Furthermore, a higher dielectric constant promotes surface charge accumulation during contact [200, 237, 238]. Owing to this dual mechanism, enhancing the dielectric constant markedly boosts triboelectric performance. Thus, tuning the dielectric properties of the triboelectric layer emerges as an effective strategy to improve the output performance of triboelectric materials. Moreover, triboelectric materials with superior wear resistance and mechanical robustness are critical for ensuring the stability and durability of TENG electrical output. Elastomers with high mechanical strength and tensile toughness stand out as promising candidates for TENGs [239, 240]. They offer distinct advantages in the design of wearable flexible devices, electronic skin, and human–machine interaction systems. However, in contrast to PENGs, which require rigorous encapsulation, TENG are susceptible to interference from environmental humidity and temperature. Therefore, engineering elastomers with excellent humidity resistance is pivotal for maintaining the stability of electrical signals.

#### Optimization of Triboelectric Power Generation Performance of Elastomers

The performance optimization of TENG necessitates systematic regulation across four key domains: material selection, structural design, energy management, and environmental adaptability. At the material level, peak performance can be realized by choosing friction material pairings that exhibit substantial disparities in electron affinity (e.g., PTFE-nylon systems). This choice should be complemented by surface micro/nano-structuring and chemical modification. These enhancements serve to elevate dielectric properties and enlarge the contact area between the positive and negative friction layers (Fig. 8c) [241]. It is noteworthy that, beyond dielectric constant regulation, enhancing polarization efficiency and charge retention capacity also constitute core strategies for improving the triboelectric effect. By modulating material molecular orientation, crystallinity, and dielectric constant to alter charge characteristics before and after corona polarization, the triboelectric properties of electret and non-electret polymers can be optimized [242]. Regarding polarization treatment techniques, the quenched polarization (QP) process enhances charge density and stability by introducing deep traps through modification of the polymer lattice structure, thereby enabling ultra-long-term storage of triboelectric charges [243]. Concerning charge compensation, research has demonstrated for the first time that charge dissipation in open air can be compensated via radical ion transfer processes, thereby achieving ultra-high charge densities [244]. Regarding enhanced charge retention capability, an acid ion sandwiching strategy stores charge by forming sandwich structures requiring high activation energy. Simultaneously, selective anion migration compensates for polarization charge dissipation, yielding positively charged triboelectric materials with superior charge retention performance [245].

Presently, TENG research still grapples with challenges such as the low efficiency of low-frequency energy harvesting and the underdeveloped large-scale fabrication processes. Future research ought to concentrate on three main directions. Firstly, develop novel functional materials with self-healing capabilities to prolong the lifespan of devices. Secondly, hybrid systems integrating TENG with other energy-harvesting technologies have been developed. By combining the piezoelectric effect [247] and magnetic induction effects [248], these systems achieve efficient energy capture across a broad spectrum. Finally, explore the application of biomimetic structures and smart responsive materials in TENG to surpass the existing performance boundaries. These innovative studies will greatly promote the practical application of TENG in areas like IoT sensors, wearable electronics, and marine energy harvesting. This thus offers new technical approaches for realizing self-powered systems.

### Dielectric Elastomer Generator

Dielectric Elastomer Generators (DEG) convert mechanical energy into electricity through cyclic stretching and releasing, with performance governed by key parameters including dielectric permittivity, breakdown strength, and elastic modulus. Energy density and conversion efficiency are enhanced through multilayer gradient architectures, pre-stretching treatments, and synchronous charge extraction circuits, positioning DEGs as efficient energy solutions for applications such as wave energy harvesting and wearable electronics.

#### Principle of Dielectric Elastomer Generators

**Fig. 9:**
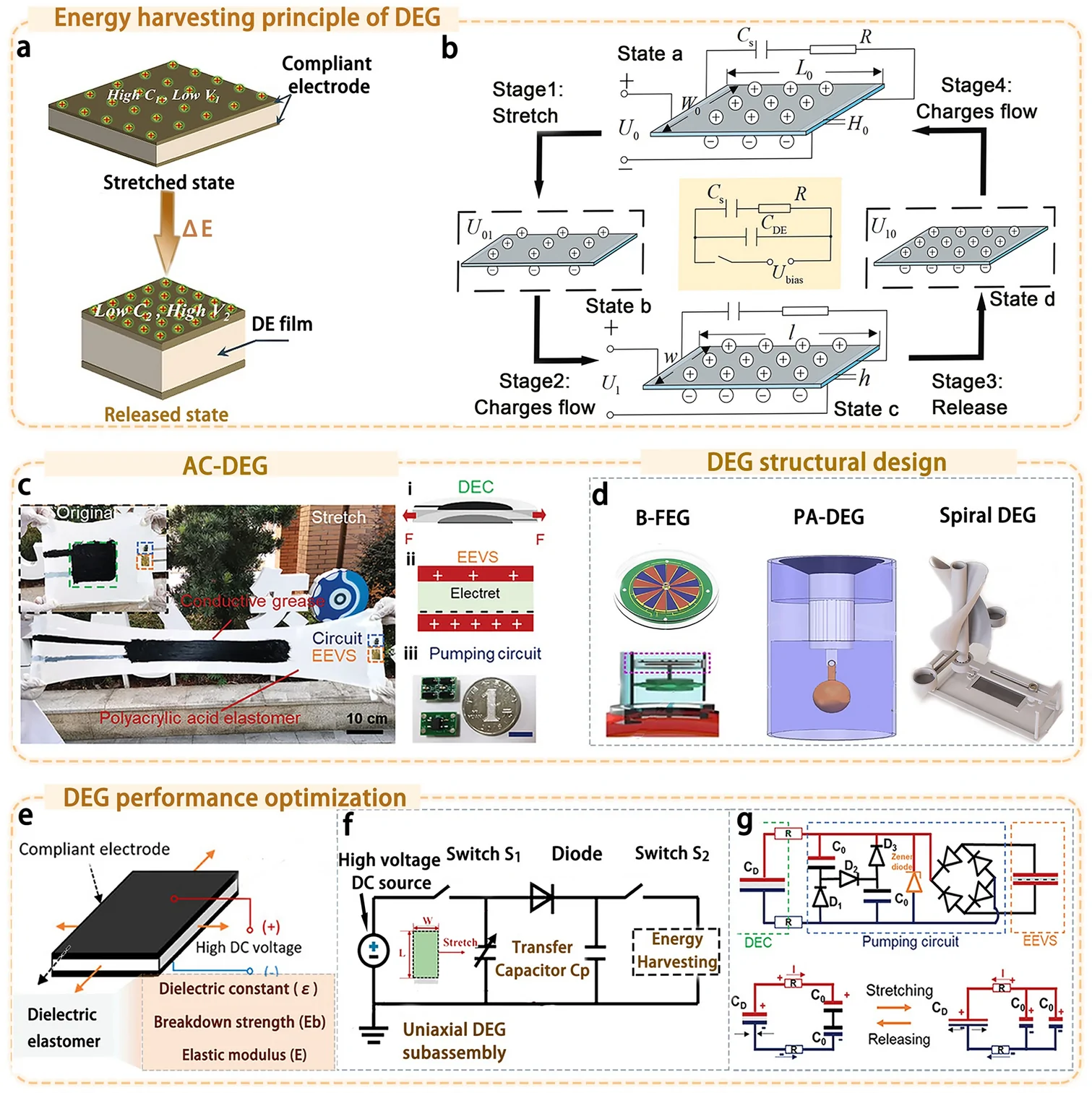
Dielectric elastic generator mechanism. **a** Energy-harvesting principle of DEG. Reproduced with permission from Ref. [251], Copyright 2024 American Chemical Society. **b** Diagram shows the complete cycle of the single-axis DEG energy conversion process, with the entire circuit model marked in the yellow shaded area. Reproduced with permission from Ref. [252], Copyright 2024 Elsevier. **c** AC-DEG consists of three parts: EC, pump loop, and EEVS. Reproduced with permission from Ref. [253], Copyright 2022 Wiley. **d** Types of DEG structural designs, including the rotary generator (B-FEG). Reproduced with permission from Ref. [254], Copyright 2023 Elsevier. Pendulum-type ring generator (PA-DEG). Reproduced with permission from Ref. [255], Copyright 2024 Elsevier. Spiral generator (Spiral DEG). Spiral generator (Spiral DEG). Reproduced with permission from Ref. [256], Copyright 2020 Elsevier. **e** Composition of the AC-DEG consists of three parts: EC, pump circuit, and EEVS. Reproduced with permission from Ref. [253], Copyright 2022 Wiley. **f** Single-axis high-voltage DC DEG circuit diagram. Reproduced with permission from Ref. [251], Copyright 2024 American Chemical Society. **g** Dual-axis AC-DEG circuit diagram. Reproduced with permission from Ref. [257], Copyright 2023 Elsevier

Relative to the material property requirements of PENG and TENG, the elastomers required for DEG impose higher demands. Specifically, dielectric elastomer (DE) materials typically require high energy density, large deformability, and high electromechanical conversion efficiency [249]. Owing to such material advantages, DEG exhibits higher electrical output power density and superior electromechanical coupling performance compared to TENG and PENG [250]. A traditional dielectric DEG is a dielectric capacitor (DEC). It consists of a DE material sandwiched between two compliant electrodes. When connected to an external high-voltage power supply, it converts input mechanical energy into electrical energy through a stretching-releasing cycle (Fig. 9a, b) [251].

First, the DEGs is stretched under an external force. This causes the area of the DE film to increase and its thickness to decrease. As a result, the capacitance of the elastomer film is enhanced. Subsequently, an external bias voltage is applied to excite the stretched DE material. Upon removal of the external force, the DE material relaxes. This leads to a decrease in its capacitance, along with an increase in voltage and electrical energy. When modeled as the simplest parallel-plate capacitor, it satisfies the assumptions of energy storage and constant charge, that is:

16 $$Q={C}_{1}{V}_{1}={C}_{2}{V}_{2}$$

Based on this, the calculation method for the theoretical electrical energy generated ($\Delta {E}_{\mathrm{theory}}$) is as follows:

17 $$\Delta {E}_{\mathrm{theory}}={E}_{2}-{E}_{1}=\frac{1}{2}{C}_{2}{V}_{2}^{2}-\frac{1}{2}{C}_{1}{V}_{1}^{2}=\frac{{\varepsilon }_{O}{\varepsilon }_{r}{A}_{1}}{2{d}_{1}}{V}_{1}^{2}\left(\frac{{A}_{1}^{2}}{{A}_{2}^{2}}-1\right)$$

where $C\left(C=\frac{{\varepsilon }_{0}{\varepsilon }_{\gamma }A}{d}\right)$ and V represents the capacitance and voltage across the DE material, respectively. ${\varepsilon }_{0}$ denotes the vacuum permittivity, ${\varepsilon }_{\gamma }$ denotes the relative permittivity of the DE material, A and d represent the effective working area and thickness, respectively, and subscripts 1 and 2 denote the ‘stretched’ and ‘released’ states of the DE material, respectively.

Based on the existing$\Delta {E}_{theory}$, two important energy-harvesting characteristics, namely energy density (w) and electromechanical conversion efficiency (η), can be calculated. The energy generated during a single cycle can be expressed as $w=\frac{\Delta {E}_{\mathrm{theory}}}{m}$, $\eta =\frac{\Delta {E}_{\mathrm{theory}}}{{W}_{\mathrm{Mech}}}$, where m represents the effective mass of the DE material and ${W}_{\mathrm{Mech}}$ represents the output mechanical work.

From the structure of traditional dielectric elastomer systems, it can be seen that conventional dielectric elastomer generators require kilovolt-level bias voltages. After each cycle of expansion and contraction, the consumed charge must be replenished by the external bias voltage, which greatly limits their practical industrial applications. To overcome this limitation, alternating current dielectric elastomer generators (AC-DEG) with passive configurations have been developed. Compared to traditional DEG, AC-DEG retain all the advantages of DEG. Meanwhile, they eliminate the need for kilovolt-level bias voltages via the use of an electret electrostatic voltage source (EEVS) and a charge pump circuit (P-Circuit) (Fig. 9c) [253]. The DEC, as the only active component, enables the AC-DEG to adopt various shapes and achieve multi-degree-of-freedom motion due to its stretchability. Meanwhile, the energy conversion efficiency and stability can be improved by optimizing the internal structural parameters (Fig. 9d). Among elastomer materials, dielectric elastomers need to exhibit excellent mechanical properties-such as elastic recovery, toughness, and tensile strength well as superior dielectric properties. All these properties are critical for the effective conversion between mechanical and electrical energy.

#### Optimization of the Power Generation Performance of Dielectric Elastomer Generators

The performance of dielectric DEG is influenced by a combination of material properties, structural design, and operating conditions. Key material parameters include dielectric permittivity (ε), dielectric strength (E_b_), and elastic modulus (E), which are interdependent (Fig. 9e). High-ε materials (e.g., BaTiO_3_/PDMS composites, ε ~ 15) can enhance charge storage capacity. However, excessive filler content (> 20 wt%) can compromise flexibility. A low E (0.1–1 MPa) allows for large deformations but may lead to a reduction in E_b_. For structural optimization, pre-stretching (100%–300%) can increase E_b_ up to 50 kV mm^−1^. Multilayer gradient designs can effectively integrate high ε with low E, while wrinkled or fiber-reinforced structures offer a balance between flexibility and durability. At the system level, load impedance must be properly matched. Synchronous charge extraction techniques can be applied, achieving energy densities of up to 0.5 J g^−1^. Additionally, resonant circuit designs can be utilized to broaden the operational frequency bandwidth (0.1–30 Hz).

The current challenge lies in resolving the inherent trade-offs among high permittivity (ε > 30), low E, and high breakdown strength (E_b_ > 100 kV mm^−1^). By designing more suitable structural configurations for DEGs and modifying their operational modes (Fig. 9f, g), together with machine learning-based optimization, energy conversion efficiency can be further improved. This advancement is expected to enable applications such as wave energy harvesting (> 1 J cm^−3^) and wearable electronics (> 1 mW cm^−2^). At the core of this progress is the multiscale synergistic optimization of materials, structures, and systems.

### Applications of Entropy-Driven Self-Assembled Cellulosic Elastomers

Entropy-driven self-assembled cellulosic elastomers exhibit versatile functionality in energy harvesting and intelligent sensing applications. These materials efficiently convert mechanical energy-such as human motion-into electricity through integrated triboelectric, piezoelectric, and dielectric transduction mechanisms. Furthermore, structural entropy changes within the material generate detectable electrical signals, enabling applications in wireless motion monitoring, non-contact sensing, and human–machine interfaces. These capabilities position cellulosic elastomers as promising components for self-powered wearable devices and adaptive intelligent systems.

#### Mechanical Energy Harvesting

**Fig. 10:**
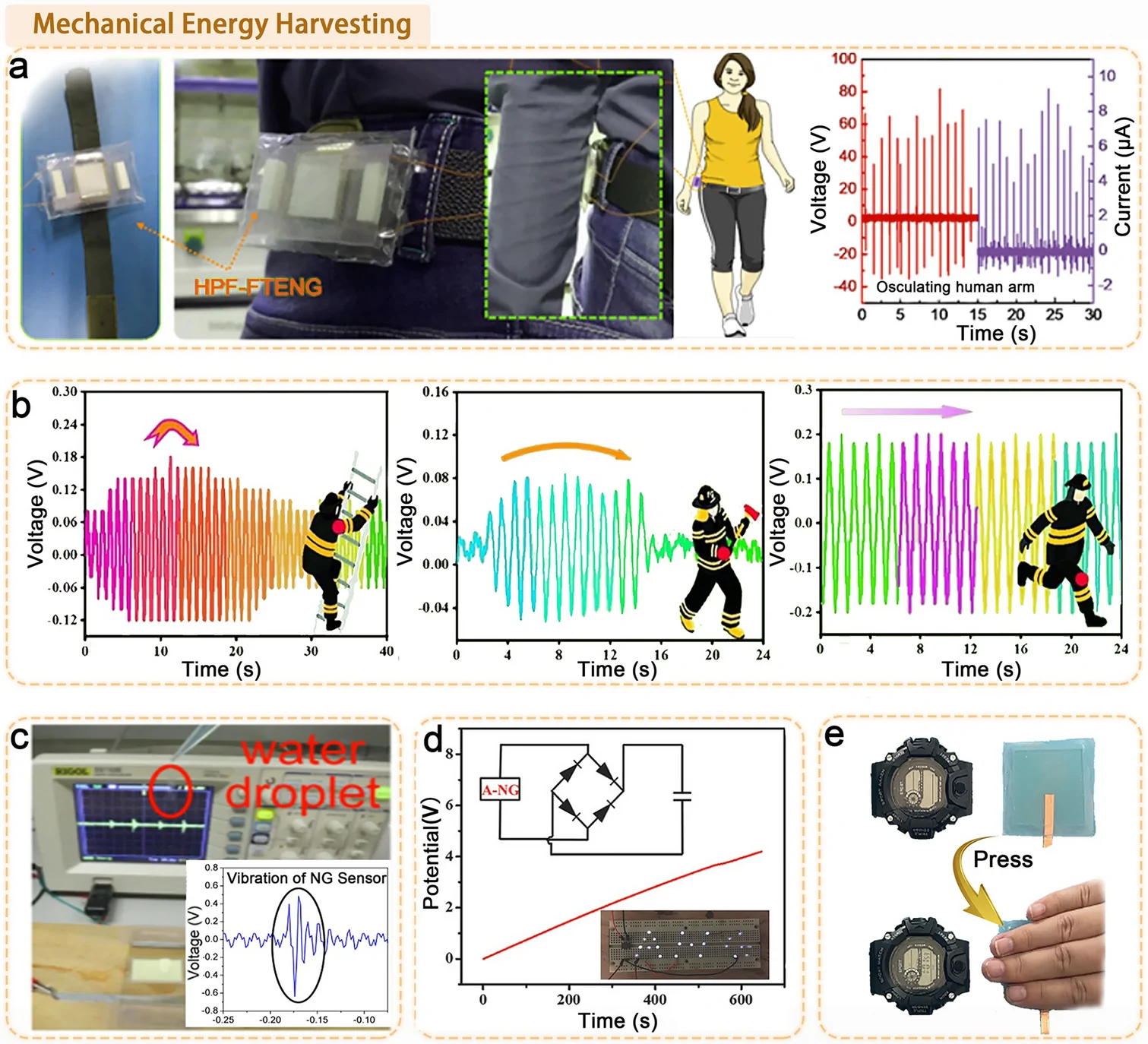
Cellulose-based elastomers for mechanical energy harvesting. **a** Schematic diagram of HPF-FTENG attached to the buttocks of human clothing and voltage and current curves collected when mechanical pressure is applied to HPF-FTENG. Reproduced with permission from Ref. [260], Copyright 2020 American Chemical Society. **b** Energy harvesting by LPCP-TENG on the human shoulder, finger, and knee. Reproduced with permission from Ref. [261], Copyright 2024 Elsevier. **c** Voltage curve collected by a micro-TENG during a water droplet fall. Reproduced with permission from Ref. [264], Copyright 2018 Elsevier. **d** Micro-TENG instantly illuminates 22 blue LEDs and the capacitor charging curve. Reproduced with permission from Ref. [265], Copyright 2018 John Wiley and Sons. **e** A watch powered by an E-TENG. Reproduced with permission from Ref. [268], Copyright 2023 John Wiley and Sons

During the process of mechanical energy harvesting, external mechanical disturbances (such as vibration and pressing) are applied to the material. This triggers an entropy-driven mechanism. The mechanism leads to dynamic reconstruction and entropy changes in the material’s internal microstructures. These microstructures include the distribution of nanofillers, molecular chain orientation, and interfacial hydrogen bond networks [258]. A triboelectric pressure sensor based on hydrophilic triboelectric elastomer and gradient microchannels enhances sensing performance through pressure-induced water-bridge modulation of the built-in electric field, ion-rich interface, and selective ion transfer, achieving simultaneous improvements in sensitivity and linearity [259]. Additionally, the FTENG, based on a porous, flexible piezoelectric film (HPF), is connected to a wearable textile belt and secured to clothing on the human buttocks. During daily activities such as walking and running, the arm may lightly contact the buttocks, inducing mechanical deformation. This enables the HPF-FTENG to efficiently harvest the generated biomechanical energy (Fig. 10a) [260]. Entropy-driven cellulose-based elastomers also show promise in the field of functional integration. Lycra fabric (LC) demonstrates significant potential in wearable TENG applications due to its high elastic recovery, shape retention, and body-hugging properties. Conductive polypyrrole (PPy) and naturally derived chitosan (CS)/phytic acid (PA), both tribo-negative materials, were sequentially applied to Lycra fabric (LC) to assemble a biodegradable and flame-retardant LPCP-TENG. The breathable and elastic LPCP-TENG can be integrated into various parts of firefighting suits to harvest mechanical energy generated by movements such as climbing, grasping equipment, and running, thereby enabling sustainable energy collection (Fig. 10b) [261].

CNFs, as a derivative material of natural cellulose, possess outstanding advantages including a high aspect ratio, high transparency, excellent mechanical strength, good flexibility, and ideal electrical properties [262, 263]. Consequently, they have become a research hotspot in the field of flexible energy storage and harvesting. However, CNF exhibits weak triboelectric properties, which can be enhanced through chemical modification. For instance, highly porous CNF/PEI aerogels were prepared via an amidation process. PEI modification alters the flexibility of CNF molecular chains and their surface charge distribution, leading to more pronounced changes in conformational entropy under mechanical stress. This endows the CNF/PEI aerogel not only with robust mechanical properties but also with exceptional triboelectric activity, significantly enhancing the output performance of the triboelectric nanogenerator (TENG). Such a TENG holds promise for harvesting energy from bodily movements such as water droplet impact. (Fig. 10c) [264].

Furthermore, electron-donating amino groups were introduced into CNF aerogel via silanization to enhance its positive polarity. The resulting CNF/CTS aerogel, based on amino-modified CNF, achieved greater molecular chain mobility through its physical pore structure. This structural feature facilitates more pronounced entropy-induced charge transfer during interfacial contact. The aerogel-derived A-NG can instantaneously illuminate 22 series-connected blue LEDs under external force and charge capacitors via a bridge rectifier, functioning as an efficient energy-harvesting power source (Fig. 10d) [265]. In addition to variations in effective contact area, crystallization induced by strain and temperature changes can also cause shifts in the triboelectric sequence. Strain-induced crystallization of molecular chains may generate ordered molecular orientation changes, thereby altering surface electron density and leading to shifts or even reversals in triboelectric polarity. Notably, while silicone rubber (Ecoflex) exhibits minimal performance variation under different strains at room temperature, strain-induced shifts in the triboelectric series become apparent at − 50 °C [266]. Hydroxypropyl cellulose (HPC), as a cellulose derivative, combines a rigid backbone, biocompatibility, and abundant hydroxyl groups [267]. It forms dense dynamic hydrogen bonds to enhance mechanical properties, while its long linear molecular chains strengthen polymer network entanglement, conferring high elasticity to eutectic gels. When incorporated into metal-salt-based eutectic solvents (MDES), the structural design anchoring cellulose to PAA chains, combined with the rapid cleavage/reconstruction of dynamic sacrificial bonds, enables the fabrication of highly resilient eutectic gels. Functioning as flexible E-TENGs, these gels harvest mechanical energy and can power digital watches through finger taps. (Fig. 10e) [268].

#### Wireless Motion Sensor

Entropy-induced charge transfer or capacitance variation can facilitate energy conversion through piezoelectric, triboelectric, or dielectric effects [269]. In self-powered sensing, environmental mechanical stimuli trigger entropy-driven reversible structural changes. These alterations, such as changes in dielectric constant, ionic conductivity, or interfacial potential difference, generate detectable electrical signal outputs, enabling sensing without external power sources [270].

**Fig. 11:**
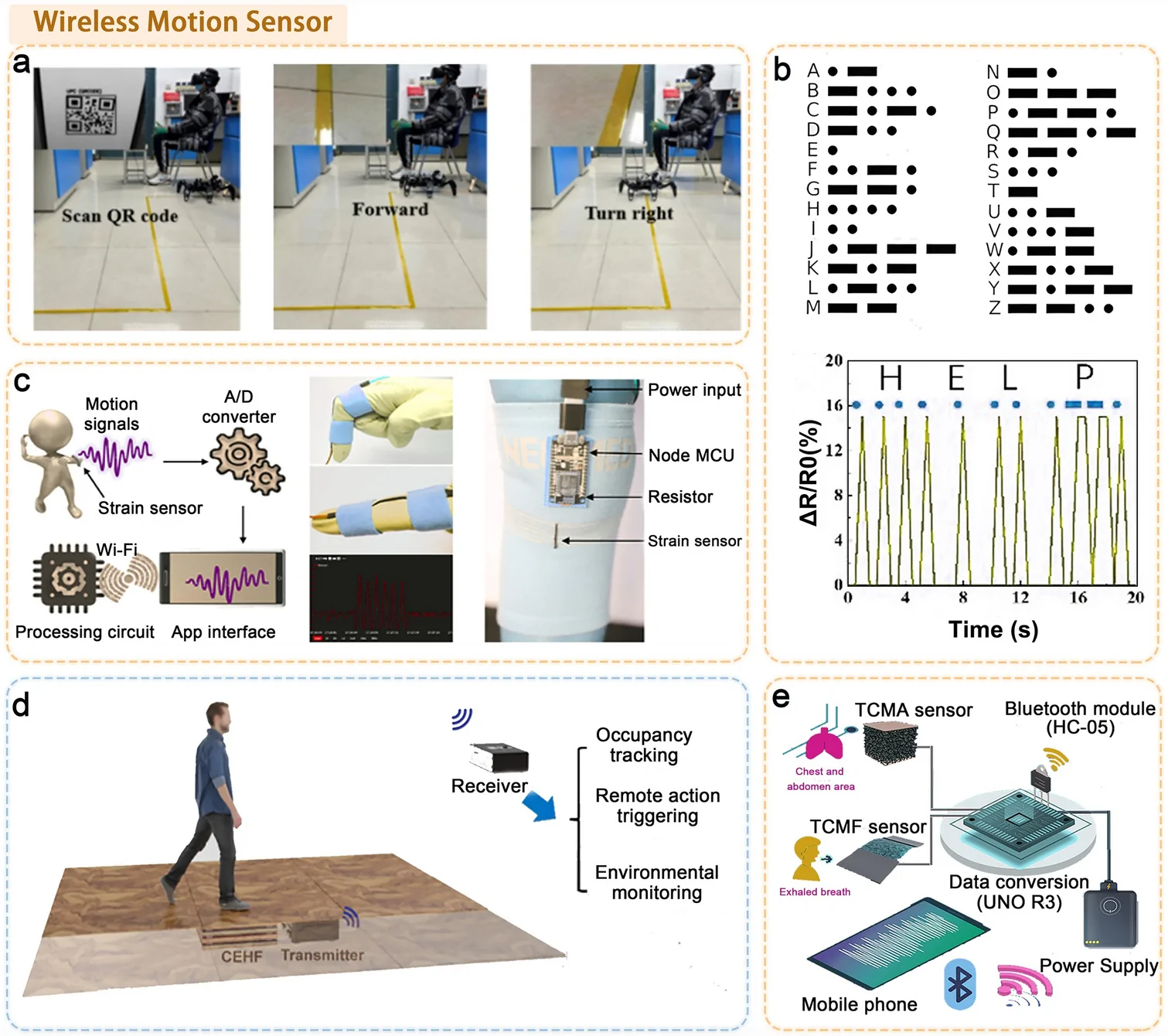
Cellulose-based elastomers for wireless motion sensing. **a** Schematic Diagram of Controlling Robot Route Patrol and QR Code Scanning. Reproduced with permission from Ref. [273], Copyright 2025 Elsevier. **b** Morse code diagram and encryption, and translation of “HELP”. Reproduced with permission from Ref. [272], Copyright 2024 Springer Nature. **c** Schematic diagram of an integrated system for wireless transmission of body movement (index finger movement, knee guard integration) sensing signals. Reproduced with permission from Ref. [274], Copyright 2025 Elsevier. **d** Schematic diagram of a real-time wireless transmission sensing system for occupancy tracking, remote action triggering, environmental monitoring, etc. Reproduced with permission from Ref. [275], Copyright 2021 American Chemical Society. **e** Schematic diagram of SAS detection using a multifunctional wireless flexible sensing platform. Reproduced with permission from Ref. [276], Copyright 2025 Elsevier

Bacterial cellulose (BC), tannic acid (TA) and LiCl were incorporated into the P(AM-co-AA) polymer network to prepare PBTL hydrogels exhibiting outstanding extensibility, adhesion, and environmental adaptability. A smart glove developed using PBTL sensors, combined with VR technology, enables wireless gesture control of a hexapod robot. (Fig. 11a) [271]. The polydopamine-modified cellulose nanofiber/polyvinyl alcohol-polyacrylamide (PCNF/PVA-PAM) composite hydrogel enables wireless signal transmission by converting mechanical deformation into resistance changes. The composite network effectively dissipates energy and enhances the hydrogel’s mechanical strength and toughness. When adhered to a finger, the composite hydrogel allows specific gestures to trigger long-distance wireless transmission of the “HELP” message (Fig. 11b) [272].

The natural rubber/cellulose nanofiber (NR/CNF) strain sensor exhibits significantly enhanced performance due to the synergistic effect between the excellent electrical conductivity of CNT/PEDOT: PSS and the mechanical reinforcement provided by CNF. During the extension and release of the index finger, wireless signals can be transmitted in real time via a mobile application, enabling simultaneous detection and processing of epidermal physiological signals [274] (Fig. 11c). Additionally, as shown in Fig. 11d, a real-time wireless transmission sensing system was developed using an energy-harvesting floor (CEHF) made from commercial cellulose materials. When a person steps on the CEHF, the converted electrical energy is used both to detect the footsteps and to power the radio frequency transmission system, allowing for the remote collection and real-time processing of pedestrian traffic data [275]. The TOCNF/MXene dual-functional sensor exhibits bimodal responsiveness to humidity and pressure. This is achieved through the synergistic integration of a three-dimensional hydrogen-bonding network (TCMF film) and a porous aerogel structure (TCMA composite aerogel). When equipped with a Bluetooth module, it enables real-time monitoring of sleep respiration waveforms, thereby addressing the limitation of single-functionality in traditional sensors (Fig. 11e) [276].

#### Intelligent Human–Machine Interaction

**Fig. 12:**
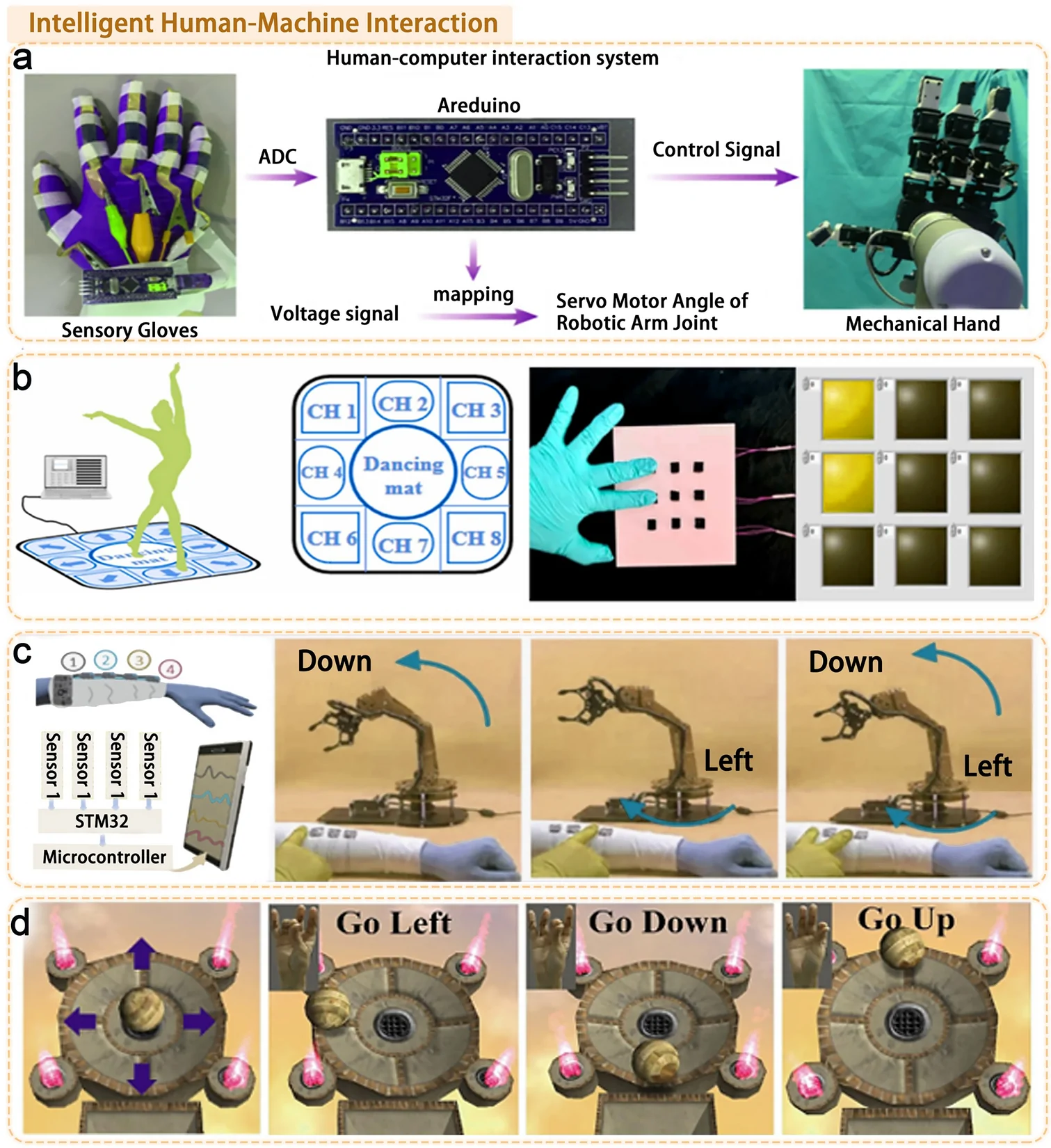
Cellulose-based elastomers for smart human–machine interaction. **a** Human–machine interaction system. Reproduced with permission from Ref. [277], Copyright 2024 Elsevier. **b** Schematic diagram of a self-powered dance mat and images of the sensor array and light bulbs when the finger touch sensor unit is activated. Reproduced with permission from Ref. [278], Copyright 2023 Elsevier. **c** Data acquisition and signal processing flowchart and human–machine control system demonstration diagram. Reproduced with permission from Ref. [279], Copyright 2024 Springer Nature. **d** Human–machine interaction based on self-powered wearable sensors used as a game keyboard to control the direction of the ball. Reproduced with permission from Ref. [280], Copyright 2024 Elsevier

The layered supramolecular conductive ionogel achieves synergistic optimization of self-healing performance and stress–strain elasticity. This is realized through an entropy-driven fracture-reconstruction mechanism of the dynamic disulfide bond network, supported by the mechanical reinforcement of a rigid hydroxypropyl cellulose framework. The incorporation of polymerizable ionic liquids enhances ionic conductivity. When used as a glove sensor, it enables accurate gesture recognition and facilitates human–machine interaction through robotic arm replication (Fig. 12a) [277].

Conductive ion gels depend on dynamic disulfide bonds for self-healing. Unlike them, MXene/TEMPO-oxidized bacterial cellulose (TOBC) double-network hydrogels achieve superior mechanical properties and self-healing capabilities. They do this through the synergistic entropy of hydrogen bonds, dynamic three-dimensional networks, and micelle interactions. Sensors fabricated from this hydrogel can be employed as wireless remote interactive devices, enabling fine motor control in robotics and virtual reality [281].

To expand the dimensions of motion trajectory monitoring, research has shifted toward multi-channel dielectric enhancement designs. The cellulose carbon nanotube aerogel TENG (CCA-TENG), due to its enhanced dielectric constant and 3D porous structure, demonstrates excellent output performance. A multi-channel human–machine interface sensor based on CCA-TENG enables motion trajectory monitoring, and a self-powered dance mat developed from it can be used to evaluate the gait mechanics distribution of dancers (Fig. 12b) [278]. Furthermore, as shown in Fig. 12c, a sleeve with four BC/polypyrole/spacer fabric (BPSF) pressure sensor channels with a layered structure can achieve human–computer interaction through the movement of each sensor channel corresponding to the controller in different directions [279]. The research extends from motion control in physical space to interactive interfaces in virtual environments. A KCNF-TENG sensor, based on kapok cellulose nanofiber film, can function as a human–machine interaction control system in computer games to control the movement of a balance ball (Fig. 12d) [280].

#### Non-Contact Signal Sensing

**Fig. 13:**
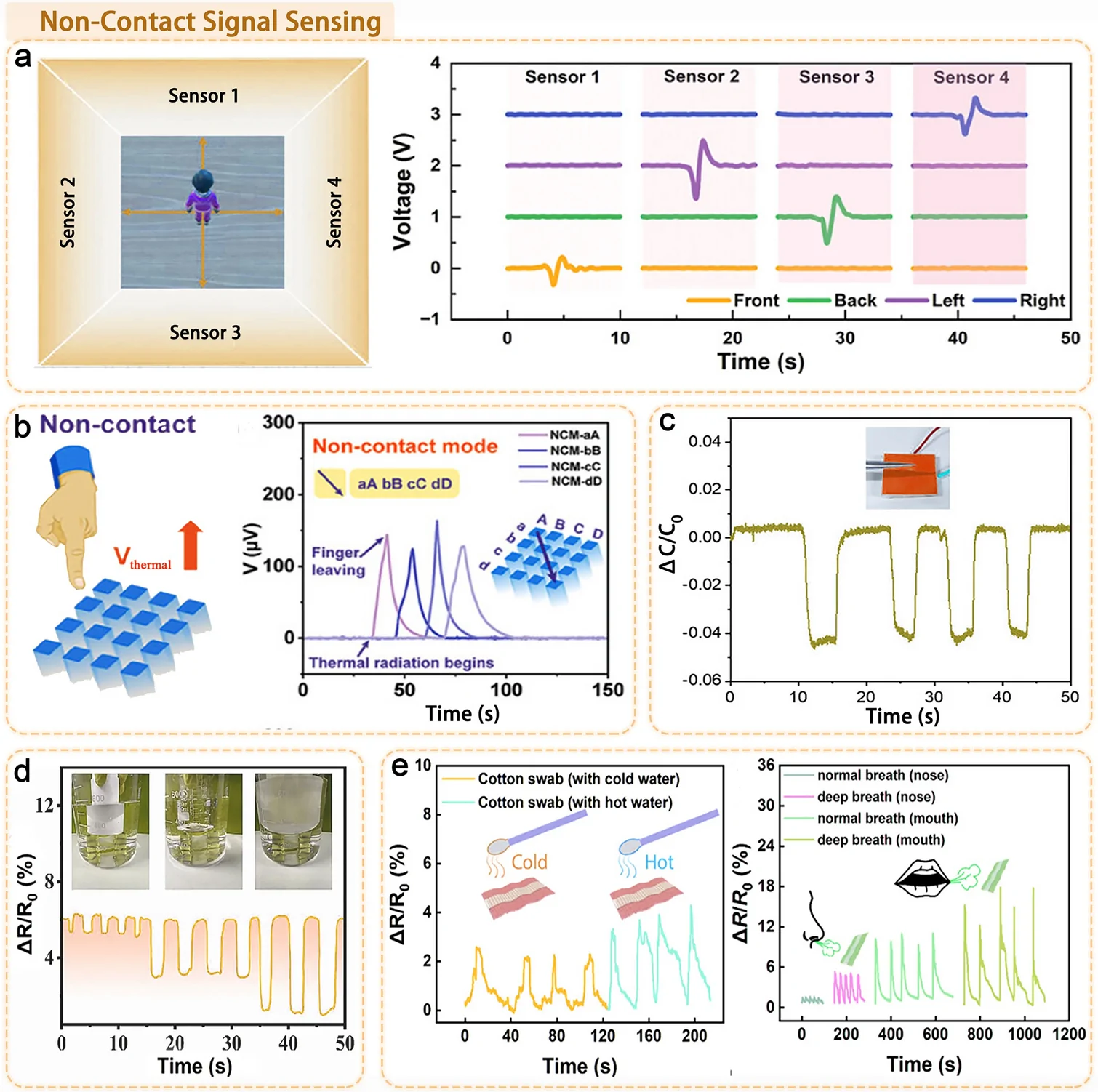
Cellulose-based elastomers for non-contact signal sensing. **a** Schematic diagram of the sensor position in the room and the output signals from the four sensors (front, back, left, and right) stimulated by non-contact movements of people in the room. Reproduced with permission from Ref. [282], Copyright 2024 John Wiley and Sons. **b** Schematic diagram of the non-contact input terminal design using a CMC sensor array and its output signals. Reproduced with permission from Ref. [283], Copyright 2024 John Wiley and Sons. **c** Plot of ΔC/C_0_ as a function of the applied non-contact touch when the tweezers hover near the device. Reproduced with permission from Ref. [284], Copyright 2025 John Wiley and Sons. **d** Sensor for identifying submerged objects in water using ΔR/R_0_. Reproduced with permission from Ref. [285], Copyright 2024 Elsevier. **e** Hot and cold cotton swabs near the sensor and the sensor’s response to different breathing patterns and humidity levels. Reproduced with permission from Ref. [286], Copyright 2025 American Chemical Society

The silk fibroin-modified carbon nanotube/bacterial cellulose/waterborne polyurethane (SSCNT/BC/WPU) gradient nanocomposite film is used to construct a Janus film by regulating the interaction between CNT and WPU. The resulting single-electrode TENG demonstrates excellent electrical output performance and enables non-contact prediction of human motion states and directions (Fig. 13a) [282]. In contrast to the Janus film, which achieves short-range non-contact sensing through electrostatic induction, the layered CNT/MXene/CNF aerogel enables non-contact sensing by utilizing thermal radiation from fingertips to generate a temperature gradient, thereby producing a thermoelectric voltage (Fig. 13b) [283]. Additionally, a parallel-plate capacitive sensor was fabricated using a large-pore, directionally layered, superelastic foam (PLA@Pulp), assembled from cellulose pulp and polylactic acid (PLA) fibers, serving as the dielectric layer to enable non-contact sensing. The extremely low dielectric constant is attributed to the high porosity of the foam and the insulating properties of PLA and pulp fibers. When tweezers approach the device, the capacitance of the sensor decreases (Fig. 13c) [284].

The research has been extended from air environments to complex underwater media. In a one-pot crosslinking process using a water-dimethyl sulfoxide binary solvent, CNC and lithium chloride (LiCl) are incorporated into a copolymer network to fabricate a conductive hydrogel (CPAMD). The CPAMD sensor demonstrates excellent non-contact sensing capabilities underwater and enables underwater alarms by distinguishing signal patterns generated by the immersion of different objects (Fig. 13d) [285]. Additionally, an electronic skin is fabricated by screen-printing a silver sensing layer between a CNF/HPC (hydroxypropyl cellulose)/PVA aerogel and a breathable polyurethane epidermal layer. The self-assembled aerogel film combines extensibility and toughness. Sensors based on this aerogel can differentiate breathing patterns, detect humidity, and achieve non-contact sensing (Fig. 13e) [286].

## Summary and Outlook

**Fig. 14:**
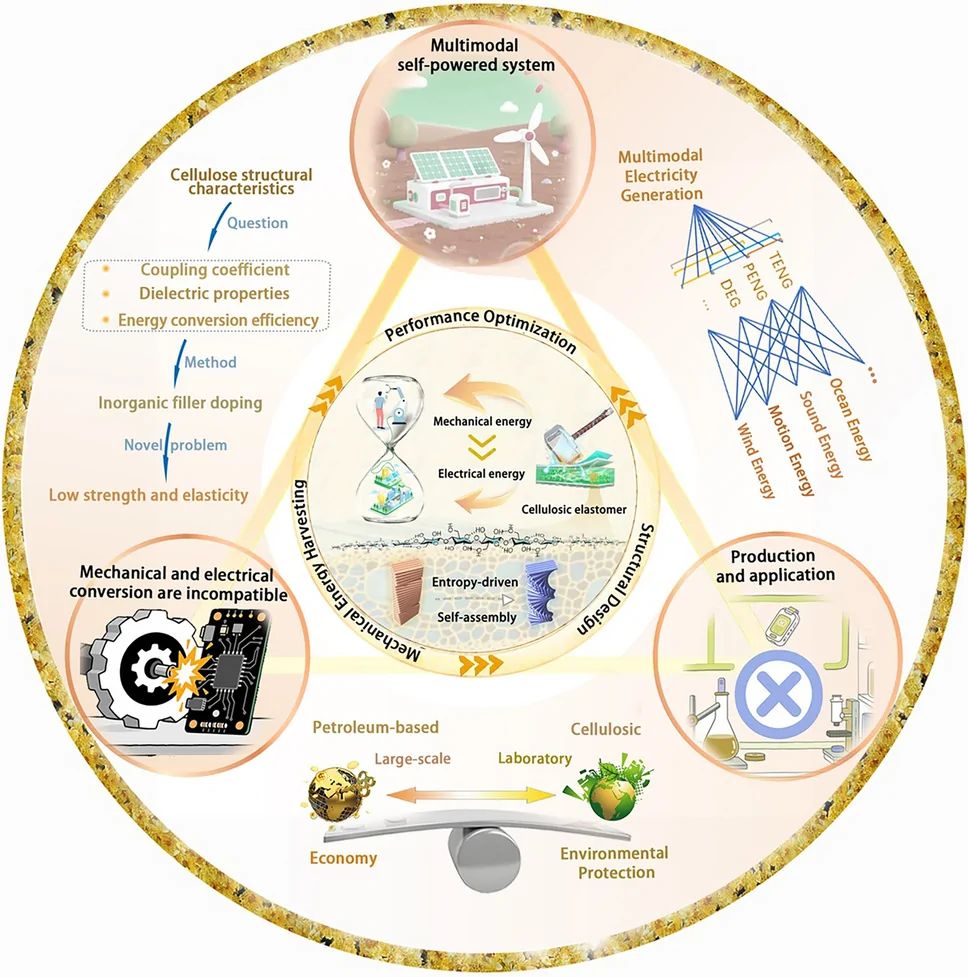
Prospects for entropy-driven self-assembled elastomers in mechanical energy harvesting and flexible sensor development

Entropy-driven self-assembly of cellulose elastomers plays a pivotal role in structural design, significantly impacting the development of novel flexible energy materials. The intrinsic connection between entropy-driven processes and self-assembly demonstrates the remarkable processability of cellulose elastomer materials through the formation of ordered structures. In cellulose elastomer design, entropy-driven approaches regulate structural transitions to tailor desired properties, serving as a key pathway to achieve electromechanical conversion efficiency. This provides a design framework for next-generation energy harvesting and sensing materials. However, challenges remain in enhancing mechanical energy conversion efficiency and advancing the practical industrial application of entropy-driven self-assembled cellulose elastomers (Fig. 14).

### Compatibility between Elasticity and Electromechanical Conversion

The electromechanical conversion performance of cellulosic elastomers is fundamentally limited by their low mechanical strength and dielectric properties. While the common strategy of incorporating high-dielectric fillers often deteriorates mechanical integrity, entropy-driven structural assembly offers a promising approach to break this trade-off. However, the intrinsically low polarity, conductivity, and polarization of cellulose still lead to inefficient charge separation and low energy conversion efficiency. Therefore, despite the unique capability of entropy-driven assembly in enhancing performance, cellulosic elastomers still significantly underperform compared to established piezoelectric materials like PVDF and PZT.

### Production and Application Dilemma

The development of well-structured cellulose-based elastomer materials typically involves high labor costs and requires relatively demanding production conditions. In contrast, petrochemical materials benefit from mature manufacturing technologies, significant economies of scale, and lower production costs. Consequently, due to cost and market constraints, much research on cellulosic elastomers remains confined to small-scale laboratory production, with manufacturing technologies still in their infancy. Global environmental initiatives are driving efforts to encourage and support the use of renewable, biodegradable natural biomass resources. These resources aim to replace petroleum-based materials. However, under current economic pressures, a challenge has emerged in balancing environmental considerations with economic viability. Achieving large-scale production of cellulosic elastomer materials while maintaining low costs and minimizing R&D requirements remains a significant challenge.

### Trend Toward Multimodal Self-Powered Integration

The power generation performance and electromechanical conversion efficiency of single-mode systems are inherently limited. The development of multimodal energy conversion technologies-such as piezoelectric, triboelectric, and dielectric elastomer generators-meets the global demand for a green and low-carbon economy, offering vast potential and significant societal value. Through using of multimodal generators, mechanical, thermal, and solar energy from the environment can be converted into electrical energy, thereby improving the efficiency of mechanical energy conversion. Achieving this demands precise process design and rigorous stability testing to meet the needs of practical production. Certain technical challenges remain. However, ongoing progress in science and manufacturing processes is expected to drive the development of multimodal elastomer materials. Such progress will contribute significantly to the sustainable growth of green energy.
